# Machine Learning-Based Security Solutions for IoT Networks: A Comprehensive Survey

**DOI:** 10.3390/s25113341

**Published:** 2025-05-26

**Authors:** Abdullah Alfahaid, Easa Alalwany, Abdulqader M. Almars, Fatemah Alharbi, Elsayed Atlam, Imad Mahgoub

**Affiliations:** 1 Department of Computer Science, College of Computer Science and Engineering, Taibah University, Yanbu 46421, Saudi Arabia; afhed@taibahu.edu.sa (A.A.); amars@taibahu.edu.sa (A.M.A.); fmhharbi@taibahu.edu.sa (F.A.); satlam@taibahu.edu.sa (E.A.); 2 Department of Electrical Engineering and Computer Science, Florida Atlantic University, 777 Glades Road, Boca Raton, FL 33431, USA; mahgoubi@fau.edu

**Keywords:** internet of things (IoT), IoT security, cybersecurity, machine learning (ML), anomaly detection, intrusion detection systems (IDSs), deep learning (DL), federated learning (FL), privacy protection, adversarial attacks

## Abstract

The Internet of Things (IoT) is revolutionizing industries by enabling seamless interconnectivity across domains such as healthcare, smart cities, the Industrial Internet of Things (IIoT), and the Internet of Vehicles (IoV). However, IoT security remains a significant challenge due to vulnerabilities related to data breaches, privacy concerns, cyber threats, and trust management issues. Addressing these risks requires advanced security mechanisms, with machine learning (ML) emerging as a powerful tool for anomaly detection, intrusion detection, and threat mitigation. This survey provides a comprehensive review of ML-driven IoT security solutions from 2020 to 2024, examining the effectiveness of supervised, unsupervised, and reinforcement learning approaches, as well as advanced techniques such as deep learning (DL), ensemble learning (EL), federated learning (FL), and transfer learning (TL). A systematic classification of ML techniques is presented based on their IoT security applications, along with a taxonomy of security threats and a critical evaluation of existing solutions in terms of scalability, computational efficiency, and privacy preservation. Additionally, this study identifies key limitations of current ML approaches, including high computational costs, adversarial vulnerabilities, and interpretability challenges, while outlining future research opportunities such as privacy-preserving ML, explainable AI, and edge-based security frameworks. By synthesizing insights from recent advancements, this paper provides a structured framework for developing robust, intelligent, and adaptive IoT security solutions. The findings aim to guide researchers and practitioners in designing next-generation cybersecurity models capable of effectively countering emerging threats in IoT ecosystems.

## 1. Introduction

Internet of Things (IoT) technology is revolutionizing industries by advancing automation, real-time data processing, and communication. In smart cities, the IoT optimizes infrastructure, including traffic management, waste disposal, and energy-efficient buildings, reducing costs and environmental impact. In healthcare, the Internet of Medical Things (IoMT) integrates wearable devices, biosensors, and remote monitoring to enhance diagnostics and chronic disease management. The Internet of Vehicles (IoV), a subset of the IoT, connects automobiles and roadside infrastructure to improve road safety, optimize traffic, and advance autonomous driving. The Industrial Internet of Things (IIoT) enables predictive maintenance and process optimization, streamlining production and logistics [1,2,3,4,5]. By 2027, the IoT is expected to exceed 41 billion connected devices [6].

Despite its benefits, the IoT presents significant privacy, trust, and security challenges. Its interconnected nature expands attack surfaces, making systems vulnerable to threats such as distributed denial-of-service (DDoS) attacks, malware, data breaches, and man-in-the-middle attacks. IoT systems also collect and store vast amounts of sensitive data, often on centralized servers, increasing privacy risks. Weak authentication mechanisms and inconsistent encryption standards further undermine trust. Medical IoT devices, for example, are frequent targets of ransomware, with potentially life-threatening consequences. Addressing these vulnerabilities requires comprehensive security frameworks that integrate trust evaluation, privacy preservation, and advanced security solutions [7,8,9,10,11].

ML and DL, key components of artificial intelligence (AI), provide innovative solutions to IoT security challenges. AI-driven anomaly detection and intrusion detection systems (IDSs) identify threats in real time. Federated learning (FL) enhances privacy by enabling local model training without exposing sensitive data. DL improves pattern recognition, distinguishing between normal and malicious activities. AI also strengthens encryption, access control, and predictive maintenance, ensuring IoT system reliability. As IoT evolves, AI integration is crucial for scalability, security, and resilience [12,13,14,15,16].

To the best of our knowledge, this is the first paper to comprehensively examine ML advancements in IoT security from 2020 to 2024. It investigates security challenges in the IIoT, healthcare, the IoV, and smart cities, identifying specific vulnerabilities and security needs. Additionally, it analyzes current ML-based solutions, highlighting limitations such as computational overhead and privacy concerns while identifying opportunities for improvement. A systematic evaluation of ML techniques, classification of security threats, and identification of trends aims to guide researchers in developing secure, intelligent IoT systems.

The main contributions of this study include:

- A comprehensive, up-to-date analysis of ML techniques for IoT security (2020–2024), identifying emerging trends and methodologies.
- Examination of key IoT security issues, including data protection, intrusion detection, privacy concerns, trust management, and threat mitigation across healthcare, the IoT, smart cities, and the IoV.
- Systematic analysis of AI methods such as ML, DL, ensemble learning (EL), transfer learning (TL), and FL in addressing IoT vulnerabilities in various domains.
- Comparative assessment of prior IoT security studies, highlighting unique contributions, gaps, and overlaps in the literature across healthcare, the IIoT, smart cities, and the IoV.
- Evaluation of challenges and limitations in applying ML techniques to IoT security, offering insights for scalable and practical security frameworks.

It is important to note that this paper does not conduct new empirical benchmarking of machine learning models. Instead, it provides a literature-based comparative analysis by consolidating and evaluating reported results (e.g., accuracy, false positive rate, computational overhead) from existing studies. This approach enables the identification of performance trends and research gaps across diverse IoT domains while acknowledging that a unified experimental evaluation remains a valuable direction for future research.

This paper is organized as follows: Section 2 reviews related surveys and identifies literature gaps. Section 4 introduces IoT architectures and applications. Section 5 discusses ML’s relevance to IoT security. Section 6 examines IoT security requirements and cyber threats across the IIoT, IoV, healthcare, and smart cities. Section 7 explores IDS for threat detection. Section 8 analyzes ML-based security solutions, focusing on DL, EL, FL, and TL in addressing security, privacy, and trust challenges. Finally, Section 9 concludes with key findings and the role of ML in strengthening IoT security.

## 2. Closely Related Surveys

This section reviews recent surveys on the application of ML and DL in IoT security. We provide a comparative analysis of their scope, methodologies, and contributions, highlighting strengths and limitations. This comparison establishes the unique position of our study in bridging existing gaps and broadening ML applications across diverse IoT domains. Table 1 systematically compares these studies, showcasing our contributions in contrast to prior surveys.

**ML Applications in IoT Security.** ML has been widely explored to address IoT security challenges [30]. While numerous surveys examine specific aspects of IoT security, they often have a limited scope, focusing on isolated applications or techniques. Our research provides a comprehensive review across multiple IoT applications, leveraging advanced ML methodologies.
**General Surveys on IoT Security.** Al-Garadi et al. [17] classify ML and DL methods based on their applications across IoT architecture layers, analyzing their security benefits and challenges. However, this study is restricted to security techniques and does not extend to other IoT applications. Hussain et al. [14] present a broad review of ML and DL in IoT security but lack specificity in addressing critical domains like healthcare, industrial IoT (IIoT), and smart cities. Ashraf et al. [18] and Jayalaxmi et al. [25] focus on ML-based intrusion detection systems (IDSs) in IoT networks but overlook broader security solutions.
**Domain-Specific Surveys in IoT.** Bharadwaj et al. [19] and Bhuiyan et al. [13] explore ML applications in the healthcare IoT (H-IoT), focusing on patient monitoring and diagnosis. However, they lack discussions on the IIoT and smart cities. Sharma et al. [20] review ML and DL applications for IIoT security but do not consider other IoT domains. Ali et al. [22] and Alalwany et al. [23] focus on ML techniques in the IoV, addressing traffic management and data security but excluding other critical IoT applications.
**Advanced ML Techniques and Trends.** Mazhar et al. [27] and Gugueoth et al. [8] review federated learning (FL) and DL for IoT security, emphasizing privacy preservation. However, these studies focus solely on security aspects, neglecting broader IoT applications. Al-Turjman et al. [24], Wu et al. [12], and Ismagilova et al. [26] examine ML in smart city IoT environments but primarily address conventional security frameworks. Pandya et al. [29] review FL’s role in smart cities, emphasizing security but limiting their analysis to this domain.
**Research Gaps and Contributions.** Unlike previous surveys, our research provides a comprehensive analysis of ML techniques for IoT security from 2020 to 2024. We address diverse security challenges, including data protection, intrusion detection, privacy, and trust management, across key IoT applications such as the IIoT, healthcare, the IoV, and smart cities. We evaluate advanced ML techniques such as DL, EL, transfer learning (TL), and FL, offering a systematic comparison of previous studies. Furthermore, Table 1 illustrates the comparative analysis, highlighting the novel contributions of our study.

## 3. Methodology

The objective of this survey is to analyze recent research and emerging trends in ML advancements for IoT security. This analysis evaluates the effectiveness of ML techniques in addressing security challenges, identifies research gaps, and highlights innovative approaches to enhance IoT resilience. It examines security issues across the IIoT, healthcare, the IoV, and smart cities, identifying their unique vulnerabilities and security needs. Furthermore, ML techniques are classified based on their specific applications within IoT security, distinguishing this survey from prior reviews. This methodology follows a structured approach similar to that of Hassan et al. [31], involving three key steps:

- **Literature Search:** A systematic search was conducted across major academic databases, including IEEE Xplore, Nature, ScienceDirect, MDPI, SpringerLink, and Google Scholar, to identify relevant research published between 2020 and 2024. Specific keywords related to ML and IoT security were used to ensure comprehensive coverage.
- **Selection:** Research papers were analyzed for key aspects such as ML techniques used, IoT applications addressed, and security challenges encountered. The search, conducted in August 2024, identified over 200 papers. Selection criteria included:
  – Publication between 2020 and 2024.
  – Relevance to ML and IoT security.
  – Use of sound methodologies in analyzing ML techniques for IoT security.
- **Data Extraction and Analysis:** Extracted data were analyzed to identify trends, research gaps, and future opportunities. ML techniques were classified based on their applications in various IoT domains, providing a structured assessment of their effectiveness in addressing security challenges in the IIoT, healthcare, the IoV, and smart cities.

Extensive research has been conducted in this area, as demonstrated in the following section.

## 4. Internet of Things: Foundations and Applications

The IoT is a transformative paradigm that interconnects devices, sensors, and systems, enabling seamless communication and data exchange across various domains. It has revolutionized industries such as healthcare, transportation, manufacturing, and urban management, fostering innovation and efficiency. This section provides an in-depth overview of the IoT, focusing on its architecture, key components, and selected applications, namely the IoV, healthcare, the IIoT, and smart cities, which were chosen due to their broad societal impact. Other important domains such as the smart home and smart grid are acknowledged but not discussed in depth due to space and scope considerations. Additionally, the challenges associated with security, privacy, and trust in IoT deployments are examined to highlight the critical need for robust protective frameworks.

### 4.1. IoT Architecture and Market Potential

The IoT comprises interconnected devices embedded with technology for communication, sensing, and interaction [32]. The global IoT market is projected to exceed $934 billion by 2033, nearly tripling its revenue from $445.3 billion in 2025 [33]. The number of connected devices is also expected to increase significantly during this period [33].

**Figure 1:**
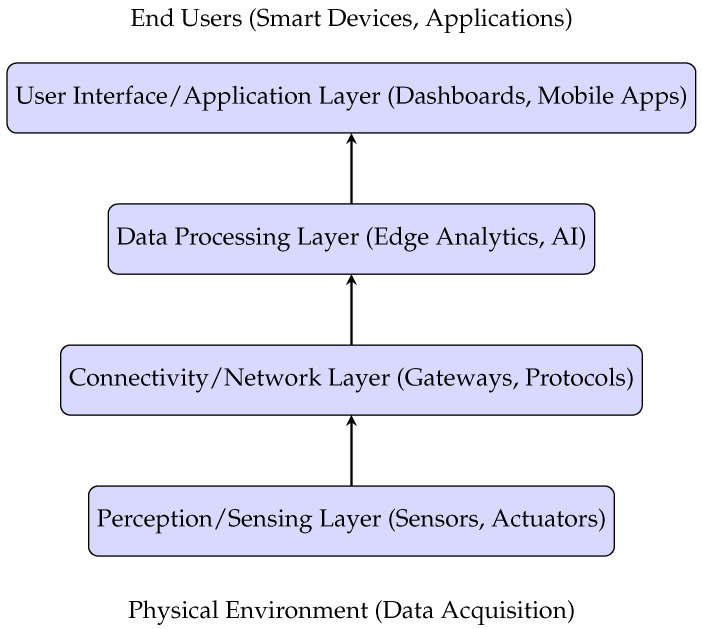
Four key layers of IoT architecture.

IoT architecture consists of four fundamental layers: the perception (sensing) layer, the connectivity (network) layer, the data processing layer, and the application (user interface) layer [34]. Figure 1 illustrates these layers, highlighting their interoperability within IoT frameworks.

Additional layers, such as computing, business, and security layers, can be incorporated to enhance specific IoT applications.

### 4.2. IoT-Related Applications

The most significant IoT applications include the IoV, healthcare IoT, IIoT, and smart city IoT. These applications are crucial due to their substantial societal impact and the distinct security, privacy, and reliability challenges they present. The IoV enhances transportation safety but involves sensitive data and mobility, creating major security concerns. The healthcare IoT improves patient monitoring but deals with critical personal data, requiring strong privacy protections and reliable performance. The IIoT optimizes industrial processes but must secure vital infrastructure to prevent disruptions and safety risks. Finally, the smart city IoT enhances urban efficiency and sustainability, but its large-scale, data-intensive nature demands robust security to protect citywide operations and residents’ data. Effectively addressing these challenges is essential for the secure and successful deployment of the IoT in these transformative applications. Brief descriptions of these IoT-related applications are provided below.

#### 4.2.1. Internet of Vehicles (IoV)

The IoV refers to a network of connected vehicles that can communicate with each other, road infrastructure, cloud servers, and personal devices [35]. It represents a subset of IoT applications and aims to enhance road safety, improve traffic efficiency, and provide a better driving experience. Here are some key aspects of the IoV:

- Typical Components of the IoV:
  – Vehicle to Vehicle (V2V): Allows vehicles to exchange information with each other about speed, direction, and location to prevent accidents and improve traffic flow.
  – Vehicle to Infrastructure (V2I): Communication between vehicles and road infrastructure, such as traffic lights, parking spaces, and toll booths.
  – Vehicle to Pedestrian (V2P): Ensures safety for pedestrians by alerting vehicles about their presence, especially in dense urban areas.
  – Vehicle to Cloud (V2C): Vehicles communicate with cloud servers for data storage, analysis, and updates, such as weather and road conditions.
- Typical Applications of IoV:
  – Traffic Management: Real-time data from IoV systems helps manage traffic flow, reduce congestion, and provide optimal routing for drivers.
  – Safety Features: The IoV enables advanced safety features, such as collision warnings, emergency braking systems, and pedestrian alerts.
  – Autonomous Driving: The IoV is a foundation for autonomous vehicles, providing data needed for safe and effective self-driving functionality.
  – Entertainment and Personalization: The IoV can enhance in-car entertainment systems, enabling personalized experiences by syncing with devices and user preferences.
  – Fleet Management: For commercial vehicles, the IoV offers tools for monitoring vehicle performance, driver behavior, and route optimization.

#### 4.2.2. Healthcare IoT

The healthcare IoT, or the Internet of Things in healthcare, refers to the interconnected network of medical devices and healthcare systems that communicate and share data over the Internet [36]. The primary goal of the healthcare IoT is to improve patient care, enhance the efficiency of healthcare services, and enable remote health monitoring. Here are some key aspects of the healthcare IoT:

- Typical Components of the Healthcare IoT:
  – Device to Device (D2D): Enables direct communication between medical devices, such as wearables and monitors, to share real-time health data.
  – Device to Hospital (D2H): Connects patient devices to healthcare providers, allowing for remote monitoring, alerts, and quick response to patient needs.
  – Device to Patient (D2P): Allows healthcare devices to provide feedback directly to patients, such as reminders for medication or alerts for abnormal health readings.
  – Device to Cloud (D2C): Healthcare devices communicate with cloud servers for storing large volumes of patient data, analytics, and updates on medical conditions.
- Typical Applications of the Healthcare IoT:
  – Remote Patient Monitoring: Allows healthcare providers to monitor patient’s health data in real time, enabling early detection of health issues and reducing hospital visits.
  – Smart Wearables: Devices such as fitness trackers and smartwatches track health metrics like heart rate, oxygen levels, and physical activity, providing insights to users and physicians.
  – Telemedicine: Enables virtual consultations and remote diagnosis, reducing the need for physical hospital visits and making healthcare accessible in remote areas.
  – Medication Management: IoT devices can remind patients to take medication, track adherence, and provide alerts for missed doses or potential drug interactions.
  – Emergency Assistance: IoT-connected devices can detect emergencies (e.g., falls, heart attacks) and automatically alert healthcare providers or emergency services for immediate response.

#### 4.2.3. Industrial IoT (IIoT)

The industrial IoT (IIoT), or the Internet of Things in industry, refers to the network of connected devices, sensors, and systems used within industrial environments to monitor, collect, and analyze data [37]. The primary goal of the IIoT is to optimize operational efficiency, enhance productivity, and enable predictive maintenance, allowing industries to reduce downtime, improve safety, and increase automation across manufacturing, energy, logistics, and other sectors. Key components and applications of the IIoT include:

- Typical Components of the Industrial IoT (IIoT):
  – Machine to Machine (M2M): Enables direct communication between industrial machines and equipment, facilitating automation and real-time data exchange for operational efficiency.
  – Machine to Cloud (M2C): Industrial machines communicate with cloud servers to store, process, and analyze large datasets, enabling predictive maintenance and advanced analytics.
  – Machine to Human (M2H): Provides interfaces for human operators to interact with machinery, allowing for monitoring, control, and adjustments based on real-time feedback.
  – Machine to Enterprise (M2E): Integrates machine data with enterprise systems, such as ERP and supply chain management, to optimize business operations and decision-making.
- Typical Applications of the Industrial IoT (IIoT):
  – Predictive Maintenance: Uses sensor data to monitor equipment health, predict failures, and schedule maintenance before breakdowns occur, reducing downtime and maintenance costs.
  – Process Automation: Enhances production processes through automated control systems, improving efficiency, quality, and consistency in manufacturing.
  – Quality Control: The IIoT enables real-time monitoring of product quality, detecting defects early and ensuring compliance with quality standards.
  – Asset Tracking: Provides real-time location and condition monitoring of assets, such as tools, machinery, and vehicles, improving asset utilization and management.
  – Energy Management: Monitors energy consumption across industrial processes, enabling efficient energy use, cost savings, and environmental sustainability.

#### 4.2.4. Smart City IoT

The smart city IoT is a broad field that integrates a variety of IoT technologies and applications to enhance urban living by making cities more efficient, sustainable, and responsive to citizens’ needs [38]. Here is a breakdown of the key components and applications of the smart city IoT:

- Typical Components of the Smart City IoT:
  – Sensor Networks: Deploy sensors across the city to monitor various parameters such as air quality, noise levels, temperature, and traffic conditions.
  – City to Citizen (C2C): Facilitates communication between city infrastructure and citizens, providing real-time information on traffic, public transportation, and city services.
  – City to Cloud (C2C): Connects urban infrastructure to cloud platforms for centralized data storage, analysis, and management of city resources.
  – City to Vehicle (C2V): Enables vehicles to interact with city infrastructure, such as traffic lights and parking systems, to optimize traffic flow and parking availability.
- Typical Applications of the Smart City IoT:
  – Smart Traffic Management: Uses real-time data from sensors and connected vehicles to manage traffic flow, reduce congestion, and optimize signal timing.
  – Waste Management: Implements smart bins with sensors to monitor waste levels, optimizing collection routes and reducing unnecessary pickups.
  – Energy Management: Monitors and manages energy consumption in city buildings, streetlights, and public facilities, enhancing energy efficiency and sustainability.
  – Public Safety: Deploys IoT-enabled surveillance and emergency response systems to enhance safety and ensure quick response to incidents.
  – Environmental Monitoring: Tracks air quality, water levels, and pollution levels to inform environmental policies and ensure the well-being of citizens.

### 4.3. Challenges in IoT-Related Applications

In the rapidly advancing Internet of Things (IoT) landscape, applications such as the IoV, healthcare IoT, industrial IoT (IIoT), and smart city IoT are transforming key areas of daily life, healthcare, industry, and urban management. However, the widespread deployment of interconnected devices in these fields introduces significant challenges related to security, privacy, and trust. As these IoT systems gather and process vast amounts of sensitive data, they become vulnerable to various security threats, raising concerns about the confidentiality, integrity, and availability of data. Privacy protection is essential, as IoT applications often collect personal or sensitive information that could be misused if exposed. Furthermore, trust among IoT entities is critical for ensuring reliable communication and preventing data manipulation. The below discussion outlines the primary challenges specific to each application, underscoring the need for comprehensive security measures, privacy safeguards, and trust management protocols tailored to the unique requirements of each IoT field.

#### 4.3.1. Challenges in the IoV

- **Security:** IoV systems are susceptible to cyberattacks such as data tampering, spoofing, and denial-of-service (DoS) attacks [23]. Ensuring the integrity and availability of communication among vehicles and infrastructure is critical to prevent accidents and maintain traffic flow.
- **Privacy:** As the IoV collects sensitive data (e.g., location, speed, driving behaviour), protecting the privacy of drivers and passengers is essential [23]. Unauthorized access to this information could lead to tracking or profiling of individuals.
- **Trust:** Trust is vital in ensuring the authenticity of data exchanged among vehicles and infrastructure. Malicious vehicles or compromised infrastructure components can inject false information, leading to potentially dangerous situations [23].

#### 4.3.2. Challenges in the Healthcare IoT

- **Security:** Healthcare IoT devices are often vulnerable to attacks that could compromise patient data and device functionality [39]. Ensuring device security against unauthorized access and maintaining system integrity is crucial to prevent data breaches.
- **Privacy:** Patient data in IoT systems is highly sensitive [39]. It requires robust measures for data anonymization and secure transmission to prevent unauthorized access and ensure compliance with healthcare privacy regulations.
- **Trust:** Trust in healthcare IoT devices and systems is necessary to ensure the reliability of health monitoring and diagnostics [40]. Patients and providers must trust the accuracy of data and alerts, particularly in critical situations.

#### 4.3.3. Challenges in the Industrial IoT (IIoT)

- **Security:** IIoT systems can be targets of industrial espionage, sabotage, or ransomware attacks, threatening operational continuity and safety [41]. Protecting industrial control systems from these threats is essential to avoid significant economic losses.
- **Privacy:** Although privacy concerns are less prominent in the IIoT compared to consumer applications, data about operational processes and employees’ interactions with machines could still pose privacy risks if mishandled [41].
- **Trust:** In the IIoT, trust is required in the reliability of sensor data and automated decision-making processes [42]. Any compromised or malfunctioning device can lead to inaccurate insights, affecting productivity and safety.

#### 4.3.4. Challenges in the Smart City IoT

- **Security:** Smart city infrastructures is exposed to a variety of cyber threats, including attacks on public services like traffic management and utilities [43]. Ensuring the resilience of smart city systems is essential to maintain public safety and prevent service disruptions.
- **Privacy:** Smart cities gather vast amounts of data from citizens, which can include location, behavioral, and personal data [43]. Maintaining citizen privacy through data minimization, anonymization, and strict access controls is a significant challenge.
- **Trust:** Trust is essential between the city and its citizens to ensure data integrity and reliability of services. Citizens must trust that their data are secure and that city systems provide accurate information for services such as traffic and public safety [43].

The aforementioned challenges highlight the need for robust security frameworks, privacy-preserving mechanisms, and trust management strategies in every single IoT application.

## 5. Machine Learning (ML): Foundations and Techniques in the IoT

**Figure 2:**
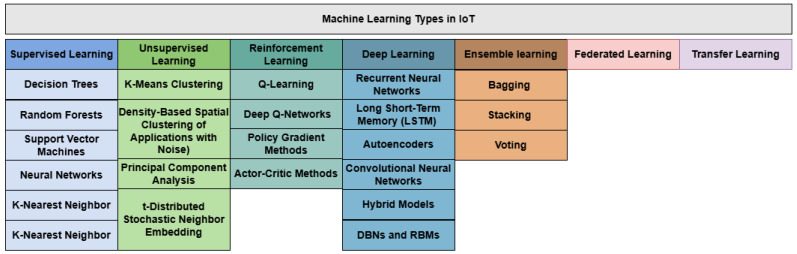
Machine learning types in IoT.

ML has emerged as a cornerstone of artificial intelligence (AI), offering unparalleled capabilities in analyzing data and automating decision-making across diverse domains. Within the context of the IoT, ML plays a pivotal role in transforming raw data into actionable insights, enhancing security, optimizing resources, and enabling intelligent decision-making. This section delves into the foundational concepts of ML, its primary types—supervised, unsupervised, and reinforcement learning—and explores advanced techniques like deep learning, ensemble learning, federated learning, and transfer learning. Emphasis is placed on the applicability of these techniques in IoT environments, showcasing their potential to address challenges such as anomaly detection, predictive maintenance, resource allocation, and cybersecurity. By leveraging ML, IoT systems are empowered to operate more efficiently and securely in dynamic, data-rich environments. Figure 2 shows ML types in IoT.

### 5.1. Supervised Learning

Supervised learning is a machine learning paradigm that involves training a model on labeled data, where each input is paired with its corresponding output. This approach is particularly valuable in IoT for tasks such as anomaly detection, where the goal is to identify patterns or instances that significantly deviate from the norm. In other words, supervised learning can be leveraged to detect and predict anomalies by learning from historical data that include both normal and anomalous instances [44,45,46,47,48].

The following are three key applications of supervised learning in the IoT: **(1) Industrial Equipment Monitoring**: Predictive maintenance of machinery in manufacturing plants [49,50,51]. **(2) Smart Home Security**: Detection of unusual activities in smart home environments. **(3) Healthcare Monitoring**: Identification of abnormal health patterns in wearable devices [52].

Examples of supervised algorithms used in IoT for anomaly detection include:

- **Decision Trees:** Used for real-time decision-making in IoT systems, such as determining whether the current state of a device is normal or anomalous based on sensor inputs [53].
- **Random Forests:** Commonly applied in IoT environments with high-dimensional data, providing robust predictions for anomaly detection in complex settings [54,55].
- **Support Vector Machines (SVMs):** Well suited for scenarios with clear class separation, such as distinguishing between normal and anomalous network traffic patterns in IoT systems [56].
- **Neural Networks:** Applied to more complex anomaly detection tasks, particularly for identifying subtle patterns in large-scale IoT data that simpler models might overlook.
- **K-Nearest Neighbors (KNN):** Effective for anomaly detection in IoT scenarios with smaller datasets, where anomalies are identified based on their proximity to known normal instances [57].

### 5.2. Unsupervised Learning

Unsupervised learning is a machine learning approach used to identify hidden patterns and structures within data that do not have labeled outputs. In the context of the IoT, unsupervised learning is particularly valuable for analyzing large datasets generated by IoT devices, where labeling data can be impractical or impossible. Unsupervised learning is particularly effective for detecting anomalies in IoT data, as anomalies often represent rare or unexpected events that are not well represented in labeled datasets. To strengthen this discussion, we have included a reference that specifically demonstrates the application of unsupervised learning techniques in the IoT. For example, Gupta and Tripathy [58] provide a comprehensive overview of unsupervised learning methods for IoT real-time data, focusing on anomaly detection and clustering. Specific unsupervised algorithms used for anomaly detection can be summarized as follows:

- **K-Means Clustering:** Used in the IoT for grouping similar device behavior, such as energy consumption patterns in smart meters. Anomalies are detected as data points that do not fit well into any cluster.
- **DBSCAN (Density-Based Spatial Clustering of Applications with Noise):** Effective for identifying anomalies in network traffic data by clustering normal behavior and detecting anomalies as noise points.
- **Principal Component Analysis (PCA):** Used in the IoT for feature reduction in sensor data, aiding in anomaly detection by highlighting unusual variance.
- **Distributed Stochastic Neighbor Embedding (t-SNE):** Useful for visualizing complex IoT data and detecting clusters of anomalous behavior.

### 5.3. Reinforcement Learning

Reinforcement learning (RL) is a branch of machine learning where an agent works with its surrounding environment to learn how to make decisions. The agent takes actions to maximize a cumulative reward over time. This paradigm is particularly effective for dynamic decision-making tasks, especially in complex and uncertain environments like the IoT. In IoT applications, RL can be utilized for various dynamic decision-making tasks, including resource allocation, energy management, and network optimization. Below are some key areas where RL enhances resource allocation in IoT systems [59]:

- **Wireless Sensor Networks (WSNs):** An RL agent can dynamically allocate bandwidth based on real-time network conditions, reducing power consumption while maintaining data integrity [60].
- **Edge Computing:** RL agents optimize task scheduling and resource allocation by learning from past decisions, thereby minimizing latency and energy consumption [61].
- **Smart Grid Management:** RL agents predict energy consumption patterns and adjust resource distribution accordingly, enhancing grid stability and reducing energy waste [62].
- **Traffic Management in Smart Cities:** RL dynamically adjusts traffic signal timings based on real-time data to optimize traffic flow and reduce congestion [63].

**How Reinforcement Learning Works in IoT [64]:** Reinforcement learning in the IoT consists of several key components:

- **Agent:** The decision-making entity that interacts with the IoT environment (e.g., a software agent managing network resources).
- **Environment:** The IoT system with which the agent interacts (e.g., a network of sensors and devices).
- **State:** The current condition of the environment, providing context for decision-making (e.g., current bandwidth usage, device battery levels).
- **Action:** A decision taken by the agent that influences the environment’s state (e.g., allocating additional bandwidth to a device).
- **Reward:** Feedback received after an action, indicating its effectiveness (e.g., improved network performance results in a positive reward).
- **Policy:** A strategy used by the agent to determine actions based on the current state (e.g., rules for resource allocation).
- **Value Function:** An estimate of the expected cumulative reward for a given state or action, guiding the agent towards optimal decisions.

**Algorithms Used in Reinforcement Learning for the IoT:** Several RL algorithms are commonly applied to IoT challenges:

- **Q-Learning:** A model-free algorithm that learns the value of actions in each state to formulate a policy. It is particularly useful in environments that are too complex to model accurately [65].
- **Deep Q-Networks (DQN):** An extension of Q-learning that integrates Deep Neural Networks to manage high-dimensional state spaces, making it ideal for complex IoT environments [66].
- **Policy Gradient Methods:** These methods directly learn a policy that maps states to actions, enabling continuous action spaces and stochastic policies [67].
- **Actor–Critic Methods:** A hybrid approach that combines value function estimation (critic) with policy learning (actor), improving stability and efficiency in complex decision-making scenarios [68].

### 5.4. Deep Learning

Deep learning is a subset of machine learning that leverages artificial neural networks with multiple layers to model complex patterns in large datasets. Its ability to automatically extract features and learn hierarchical representations makes it highly effective for processing large-scale IoT data. In the context of the IoT, deep learning is particularly useful for enhancing security measures, as it can analyze vast amounts of data from various devices to detect anomalies, identify threats, and improve overall system resilience.

Deep learning can significantly enhance IoT security by providing robust solutions for threat detection, anomaly detection, and data protection. Here are some key applications and techniques used in IoT security:

- **Anomaly Detection:** Anomaly detection is critical for identifying irregular activities or deviations from normal behavior, which could indicate security threats such as intrusions or malware attacks as [69]. Several deep learning techniques such as Recurrent Neural Networks (RNNs), Long Short-Term Memory (LSTM) networks and Autoencoders can used for anomaly detection. RNNs and LSTM can learn patterns of normal behavior in IoT data streams and detect anomalies, such as unexpected spikes in network traffic or unusual device activity, which may indicate security breaches. Furthermore, Autoencoders can be used to detect anomalies in network traffic data, device behavior logs, or sensor readings, helping to identify potential security threats.
- **Intrusion Detection Systems (IDSs):** Deep learning can enhance traditional intrusion detection systems by providing more accurate and adaptive threat detection [25,70]. Convolutional Neural Networks (CNNs) can classify network traffic patterns and detect intrusions based on anomalies in data packets, improving the detection of sophisticated cyberattacks. Moreover, Hybrid models, such as combining CNNs with LSTMs, can be used for real-time intrusion detection in IoT networks, leveraging both static and dynamic data features.
- **Malware Detection:** Deep learning can be utilized to identify and prevent malware attacks on IoT devices by analyzing code or behavior patterns. For instance, Deep Belief Networks (DBNs) and Restricted Boltzmann Machines (RBMs) can analyze binary code or network behavior to detect malware signatures or suspicious activities, offering proactive protection against cyber threats [71].
- **Authentication and Access Control** Deep learning can enhance authentication mechanisms by analyzing behavioral biometrics or device usage patterns [72]. RNNs, for example, can be used to detect unusual login attempts or access patterns, providing an additional layer of security through behavior-based authentication.

### 5.5. Ensemble Learning

Ensemble learning is a machine learning technique that combines multiple models to improve the overall performance and accuracy of predictions. By leveraging the strengths of diverse models, ensemble learning can reduce variance and bias, leading to more robust and reliable results. This approach is particularly useful in complex and dynamic environments like the IoT, where data can be noisy and unpredictable. Ensemble learning is commonly employed in the IoT for a variety of tasks due to its effectiveness in managing diverse and complex datasets. Key applications include (1) anomaly detection, where it identifies unusual patterns in network traffic or sensor readings to uncover potential security threats or equipment failures; (2) predictive maintenance, which utilizes ensemble models to analyze sensor data from industrial machinery to forecast and mitigate possible breakdowns; (3) energy management, optimizing energy consumption in smart grids or smart homes by predicting peak usage times and adjusting energy distribution accordingly; and (4) fault diagnosis, where it helps diagnose issues in connected vehicles by analyzing data from multiple sensors and subsystems [73].

There are several popular ensemble learning techniques, each with its own approach to combining models as follows:

- **Bagging (Bootstrap Aggregating):** Random Forest is a well-known bagging technique where each model is a Decision Tree. It is widely used in IoT applications for tasks like anomaly detection and sensor data classification due to its robustness and accuracy [74].
- **Boosting:** AdaBoost, Gradient Boosting, and XGBoost are popular boosting algorithms used in the IoT for improving predictive performance in areas such as network intrusion detection and fault diagnosis [75].
- **Stacking (Stacked Generalization):** Stacking can be used in the IoT to integrate different types of models (e.g., Decision Trees, neural networks, and Support Vector Machines) to enhance predictive accuracy and capture complex patterns in the data [76,77].
- **Voting:** Simple voting ensembles are used in IoT applications for quick and straightforward model combination, improving prediction stability and accuracy [78,79].

### 5.6. Federated Learning

Federated learning is a decentralized approach to machine learning that enables model training across multiple devices or locations while keeping the data localized. This method allows the development of models without centralizing the data, which enhances privacy and security. Federated learning is particularly beneficial in the context of IoT, where data are generated across a vast network of connected devices. Federated learning is well suited for various IoT applications, including smart home devices, healthcare and wearables, autonomous vehicles, and the industrial IoT. This is due to its ability to handle distributed data while preserving privacy. The main idea behind federated learning can be summarized as follows [80]:

- **Decentralized Data:** Unlike traditional machine learning, where data are collected and processed centrally, federated learning keeps the data on local devices. Only the model updates (gradients) are shared with a central server [81].
- **Privacy and Security:** Since the data never leave the local devices, federated learning offers enhanced privacy and security, reducing the risk of data breaches [82].
- **Collaborative Learning:** Multiple devices collaboratively contribute to the model’s learning process, improving its generalization across diverse data sources [83].
- **Communication Efficiency:** Federated learning reduces the need to transmit large datasets over the network, focusing instead on model updates, which are typically smaller [84].

### 5.7. Transfer Learning

Transfer learning is a machine learning approach in which a model created for one task serves as the foundation for a model focused on a different task. This approach is especially useful when there is limited data available for the new task, as it leverages the knowledge gained from the original task. In the context of IoT security, transfer learning can significantly improve efficiency by adapting pre-trained models to quickly and effectively address security challenges. Transfer learning can enhance IoT security by efficiently adapting models for various tasks, such as intrusion detection, anomaly detection, and malware classification as follows [85]:

- **Intrusion Detection:** By leveraging models pre-trained on similar security datasets, organizations can rapidly deploy effective intrusion detection systems with reduced need for extensive labeled IoT-specific data [86,87].
- **Anomaly Detection:** Transfer learning allows for quick adaptation to various sensor environments, enabling real-time anomaly detection without the need for extensive data collection and labeling.
- **Malware Classification:** This approach accelerates the development of malware detection systems, enhancing their ability to recognize and respond to evolving threats.

## 6. Security Requirements and Cyberattack Landscape in IoT Applications

The rapid adoption of IoT technologies across smart cities, healthcare, connected vehicles, and the industrial IoT (IIoT) has revolutionized digital ecosystems, enhancing efficiency, automation, and connectivity. However, reliance on IoT devices introduces significant security risks, necessitating robust security measures. A 2020 study revealed that 98% of IoT device traffic is unencrypted, exposing sensitive data, while 57% of IoT devices are vulnerable to medium- or high-severity attacks [26,88].

This section outlines the security requirements essential for data integrity, privacy, and operational reliability, alongside prevalent cyberattacks targeting IoT systems. Understanding these threats and countermeasures is crucial for developing adaptive defense strategies.

### 6.1. Security Requirements

While IoT security requirements have been extensively studied [23,89,90,91,92], existing research often lacks application-specific insights. This section addresses the unique security challenges across different IoT domains.

#### 6.1.1. Smart Cities

Smart cities utilize the IoT to optimize urban infrastructure but require stringent security measures:

- **Data Integrity and Authenticity:** Ensuring the accuracy and security of data used in traffic management, energy distribution, and public safety is critical [93,94].
- **Access Control:** Robust authentication mechanisms, including multi-factor authentication and attribute-based access control, restrict unauthorized access [95,96,97].
- **Privacy Protection:** Privacy-preserving techniques, such as anonymization and blockchain-based encryption, safeguard citizen data [98,99,100,101].
- **Resilience Against Attacks:** Systems must withstand and recover from distributed denial-of-service (DDoS) and other cyberattacks through redundancy and incident response planning [102,103].

Smart cities prioritize the protection of critical urban data and operational continuity against cyberattacks. Their primary security requirements focus on maintaining data authenticity, enforcing stringent access controls, protecting citizen privacy, and ensuring resilience against service disruptions.

#### 6.1.2. Healthcare IoT

The IoT in healthcare enhances patient care but requires stringent security measures:

- **Patient Data Confidentiality:** Encryption safeguards patient records and ensures compliance with privacy regulations such as the HIPAA and GDPR [104,105,106,107].
- **Device Authentication:** Secure authentication protocols prevent unauthorized access to medical devices [108,109,110].
- **Data Accuracy:** Ensuring data integrity is critical for accurate diagnoses and treatments [111,112].
- **Regulatory Compliance:** Compliance with healthcare regulations mitigates legal and ethical risks [13].

The healthcare IoT focuses on safeguarding sensitive medical data and device security. Key requirements include patient data confidentiality, secure device authentication, assurance of data accuracy, and strict adherence to healthcare regulations to prevent legal and safety risks.

#### 6.1.3. Connected Vehicles

Connected vehicles rely on the IoT for navigation and safety but face cybersecurity risks:

- **Secure Communication:** Encryption of Vehicle-to-Vehicle (V2V) and Vehicle-to-Infrastructure (V2I) communications prevents unauthorized access [113].
- **Firmware Integrity:** Cryptographic verification ensures secure Over-the-Air (OTA) firmware updates [114,115].
- **Access Control:** Biometric and advanced authentication methods prevent unauthorized access [113].
- **Real-Time Threat Detection:** Anomaly detection systems mitigate threats like spoofing and jamming [116,117].

Connected vehicles demand robust communication encryption, firmware integrity, and real-time threat detection to maintain operational safety. Security measures focus on preventing communication compromise, unauthorized access, and ensuring resilience against spoofing and jamming attacks.

#### 6.1.4. Industrial IoT (IIoT)

The IIoT improves industrial efficiency but introduces security challenges:

1. **Operational Continuity:** Robust security protocols mitigate ransomware and sabotage risks [118].
2. **Data Integrity:** Preventing data manipulation ensures industrial process reliability [119].
3. **Access Control:** Strict authentication and authorization prevent unauthorized access [120,121,122,123].
4. **Incident Response:** Effective response plans minimize damage from security breaches [124].

IIoT applications emphasize securing operational continuity and data integrity. Key requirements include protecting against ransomware attacks [9], ensuring secure access control for critical systems, and implementing effective incident response strategies to limit potential damages [125].

### 6.2. Common Threats and Cyberattack Types

IoT systems are susceptible to various cyberattacks that exploit vulnerabilities in their design, communication, and operation. This section categorizes and explains prevalent attacks and their impacts on smart cities, healthcare, connected vehicles, and the industrial IoT (IIoT).

#### 6.2.1. DoS and DDoS Attacks

DoS and DDoS attacks overwhelm IoT networks or devices with excessive traffic, rendering them inoperable. Attackers use compromised devices (botnets) to launch these attacks, exploiting weak authentication and limited processing capabilities. Notable examples include Mirai [126] and Gafgyt [127].

**Impact in Each Application:**
  - **Smart City:** Disruptions in traffic management, energy distribution, and public safety due to DDoS attacks can cause severe consequences. The IDCPRO-DLM model achieved 98.53% accuracy in detecting such attacks [86].
  - **Healthcare:** DDoS attacks disrupt real-time monitoring and patient care. Federated Generative Adversarial Network (GAN) models achieved 92.98% accuracy in mitigating these attacks [128].
  - **Connected Vehicles:** Attacks can impair Vehicle-to-Vehicle (V2V) and Vehicle-to-Infrastructure (V2I) communication, increasing accident risks. Long Short-Term Memory (LSTM) models achieved 99.5% accuracy in detection [129].
  - **IIoT:** Disruptions in manufacturing and supply chains are major concerns. A stacked ensemble model achieved 99.7% accuracy in detecting DDoS threats [130].

DoS and DDoS attacks present critical threats across all IoT applications by disrupting essential services, compromising safety, and impeding operational functionality. Mitigation techniques such as machine learning-based detection models [131,132] have shown high accuracy across domains.

#### 6.2.2. Data Breaches

Data breaches occur when attackers exploit vulnerabilities to access sensitive information, leading to identity theft, espionage, or financial fraud.

**Impact in Each Application:**
  - **Smart City:** Breaches expose citizen data and critical infrastructure information. Privacy-preserving frameworks using blockchain enhance security [133].
  - **Healthcare:** Exposure of patient data can violate privacy laws such as the Health Insurance Portability and Accountability Act (HIPAA). ML models achieved over 95% accuracy in detecting breaches [134].
  - **Connected Vehicles:** Data breaches compromise passenger privacy and security. Federated learning (FL) models improved breach mitigation with an 82.6% coverage rate [135].
  - **IIoT:** Breaches expose industrial secrets and disrupt operations. FL with deep reinforcement learning (DRL) achieved high accuracy in breach detection [136].

Data breaches severely impact privacy, confidentiality, and competitive advantage across IoT domains. Defense strategies such as blockchain-based frameworks, federated learning [137,138,139], and ML-enhanced detection models [140] are vital to mitigate breach risks. In particular, blockchain-based secure storage mechanisms, such as BSMD [141], have been proposed to protect large-scale spatio-temporal IoT data, offering decentralized security, immutability, and efficient access control against data breaches.

#### 6.2.3. Unauthorized Access

Unauthorized access occurs when attackers bypass authentication to control IoT devices or networks. Weak credentials and poor access control facilitate these breaches.

**Impact in Each Application:**
  - **Smart City:** Attackers may control street lighting or public utilities. Kernel Principal Component Analysis (KPCA) with VGG-16 achieved 96% accuracy in detecting unauthorized access [142].
  - **Healthcare:** Unauthorized control of medical devices can endanger patients. Federated GAN models achieved 92.98% accuracy in securing the healthcare IoT [128].
  - **Connected Vehicles:** Unauthorized access can compromise vehicle safety. Transfer learning (TL) with Convolutional Neural Networks (CNNs) reached 99.25% detection rates [143].
  - **IIoT:** Attackers can halt production and disrupt supply chains. Hybrid LSTM–Deep Neural Network (DNN) models achieved 99.94% accuracy in IIoT security [144].

Unauthorized access threatens control integrity and safety across IoT systems. Advanced authentication mechanisms and AI-driven detection models [145,146] significantly enhance resilience against access breaches.

#### 6.2.4. Poisoning Attacks

Poisoning attacks involve injecting malicious data into ML training datasets, degrading model performance and introducing vulnerabilities.

**Impact in Each Application:**
  - **Smart City:** Compromised ML models affect traffic and resource management. Blockchain-based validation systems mitigate such risks [133].
  - **Healthcare:** Poisoned data can lead to incorrect diagnoses. FL with Secure Multi-Party Computation (SMPC) achieved 97.69% accuracy in mitigating these attacks [147].
  - **Connected Vehicles:** Poisoned navigation data reduce communication reliability. TL models improved robustness against such attacks [148].
  - **IIoT:** Corrupted data impact industrial decision-making. FL with Generative Adversarial Networks (GANs) improved detection accuracy by 8% [149].

Poisoning attacks compromise the reliability of AI-based decision-making across IoT systems. Countermeasures like blockchain validation [150], secure federated learning [137,138,139], and robust model training techniques [151] are critical defenses.

#### 6.2.5. Malware and Botnets

Malware exploits IoT vulnerabilities to disrupt operations or steal information. Botnets, networks of infected devices, are used for large-scale attacks.

**Impact in Each Application:**
  - **Smart City:** Malware disrupts infrastructure, as seen with the Mirai botnet [126]. RF-RBN models achieved 95% accuracy in botnet detection [152].
  - **Healthcare:** Malware in medical devices can compromise patient care. LSTM–Decision Tree (DT) models achieved 0.96 F1-score in detecting threats [153].
  - **Connected Vehicles:** Malware disrupts vehicular communication and safety features. ML models achieved high precision in botnet detection [154].
  - **IIoT:** Malware can halt production and steal industrial data. Hybrid LSTM-DNN models achieved 99.94% accuracy in botnet detection [144].

Malware and botnet attacks cause massive disruptions and data theft across IoT applications. AI-based detection frameworks and endpoint protection strategies [155] are essential to defend against such threats.

## 7. Intrusion Detection Systems (IDSs) in the IoT: Mechanisms, Techniques, and Challenges

Intrusion detection systems (IDSs) are crucial in cybersecurity, protecting networks and systems from malicious activities. In IoT environments, deploying IDSs is challenging due to the vast amounts of interconnected devices generating diverse and sensitive data. This section provides an overview of IDSs in the IoT, covering fundamental principles, components, classifications, detection mechanisms, and advanced techniques, including signature-based, anomaly-based, and machine learning-driven approaches. Additionally, key challenges such as privacy concerns, scalability issues, and the need for explainable models are discussed, highlighting the evolution and future potential of IDSs in IoT security.

### 7.1. Background and Overview

**Figure 3:**
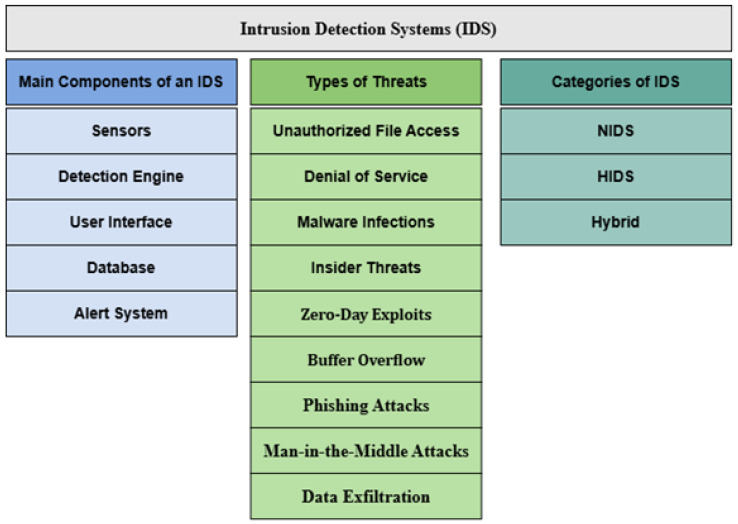
Overview of intrusion detection systems (IDSs).

The IDS dates back to the 1980s, when James P. Anderson proposed a security monitoring tool to detect unauthorized access [156]. The IDS aims to identify, analyze, and respond to malicious activities or policy violations, ensuring information systems’ integrity, confidentiality, and availability [157,158,159]. Figure 3 illustrates IDS components and functions.

The IDS plays a key role in cybersecurity, detecting various cyber threats such as:

- **Unauthorized File Access:** Detecting unauthorized access to sensitive files.
- **Denial of Service (DoS):** Identifying DoS attacks that disrupt network availability.
- **Insider Threats:** Recognizing potential abuse by authorized users.
- **Zero-Day Exploits:** Detecting unknown vulnerabilities and attacks.
- **Buffer Overflow:** Identifying software vulnerabilities from buffer overflows.
- **Malware Infections:** Detecting malicious software, including viruses, worms, and Trojans.
- **Phishing Attacks:** Identifying deceptive emails or websites aimed at stealing sensitive information.
- **Man-in-the-Middle Attacks:** Detecting interception and potential alteration of communications.
- **Data Exfiltration:** Preventing unauthorized transfer of sensitive data.

An IDS comprises five primary components: (1) sensors that collect network packet data, (2) a detection engine analyzing data for intrusions, (3) a user interface for administrator management, (4) a database storing event data and configurations, and (5) an alert system notifying administrators of threats.

IDSs can be categorized into four main types: network intrusion detection systems (NIDSs), host-based intrusion detection systems (HIDSs), application protocol-based intrusion detection systems (APIDSs), and hybrid IDSs. **NIDSs** monitor network traffic for signs of threats across the network. **HIDSs** operate on specific endpoints, detecting local anomalies. **APIDSs** focus on specific application protocols, identifying threats undetectable by traditional NIDSs or HIDSs. **Hybrid IDSs** integrate features of NIDSs and HIDSs for comprehensive security.

### 7.2. Intrusion Detection Mechanisms for the IoT

**Figure 4:**
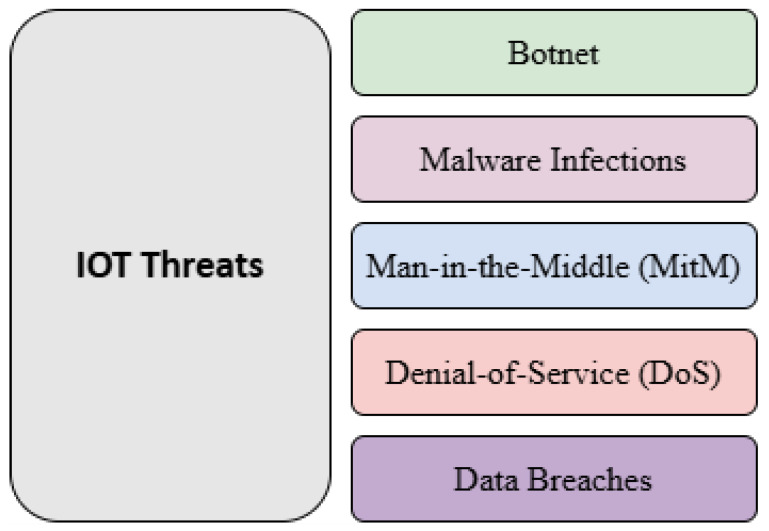
Types of IoT Threats.

The IDS plays a crucial role in securing IoT environments by enabling early threat detection and mitigation. IoT systems are vulnerable to attacks such as botnets, malware infections, MitM, DoS, and data breaches (Figure 4). IDS techniques in the IoT can be classified into four categories:

- **Signature-Based Detection:** Identifies known threats by matching activity with stored attack signatures.
- **Anomaly-Based Detection:** Detects outliers by comparing activity against normal behavioral baselines.
- **ML-Based Detection:** Uses ML algorithms to analyze data and detect complex attack patterns.
- **Hybrid Approaches:** Combine signature-based and anomaly-based methods for improved detection.

Several IDS techniques have been proposed in IoT research. For anomaly-based IDSs, Passban protects IoT devices from threats [160], while a two-level hybrid IDS identifies DDoS, DoS, and other anomalies [161]. CNN-based IDS effectively detects abnormal network behavior [162]. Thamilarasu et al. developed an IDS using deep learning to identify multiple IoT threats [163]. Shurman et al. integrated signature-based and anomaly-based IDS for early DoS attack detection [164]. Artificial Neural Networks (ANNs) were also utilized to classify threats such as DoS, Probe, U2R, and R2L [165]. Bhavsar et al. combined the Pearson Correlation Coefficient with Convolutional Neural Networks (PCC-CNNs) for IDSs, achieving high accuracy [166]. Bacha et al. applied the Kernel Extreme Learning Machine (KELM) to detect IoT threats with 99.4% accuracy [167].

For signature-based IDSs, Sheikh et al. developed a lightweight IDS for edge IoT devices [168]. Ioulianou et al. integrated centralized and distributed IDS modules [169]. Farooq et al. proposed an IDS for 6G IoT networks [170]. Otoum et al. introduced AS-IDS, combining anomaly-based and signature-based detection [171]. A hybrid IDS using behavior-based detection was evaluated with the SWaT dataset [172]. Thankappan et al. designed a centralized IDS to detect MitM attacks with a 90% true positive rate [173]. A distributed IDS was also developed for wireless environments [174].

Machine learning techniques have also been extensively applied in IDS research. Bagui et al. employed Logistic Regression (LR), Support Vector Machine (SVM), and Random Forest (RF) to detect IoT botnet traffic [175]. Altulaihan et al. used Decision Tree (DT), RF, k-Nearest Neighbors (kNN), and SVM to mitigate DoS attacks [176]. Verma et al. evaluated seven ML classifiers for IDSs [177]. Deep learning methods have also been used, achieving over 99% accuracy in detecting IoT threats [178]. Honeypots combined with ML have been utilized for botnet detection [179], demonstrating high accuracy.

These studies highlight the significance of IDS in securing IoT environments and the growing adoption of AI-driven solutions for enhanced threat detection. Table 2 summarizes different IDS approaches reported in the literature.

### 7.3. Challenges

Based on the analysis of the existing literature [182,183,184,185], this section provides a summary of the key challenges associated with implementing IDSs in IoT environments.

- **Emerging and Sophisticated Attacks:** IoT networks comprise numerous interconnected devices, making them at risk of emerging and more complex cyber attacks. The current IDS solutions lack the capability to accurately identify and understand emerging attack patterns, making them less effective in detecting new types of attacks. Therefore, developing innovative and lightweight IDS solutions is essential to enhance detection accuracy and mitigate emerging threats.
- **Privacy and Confidentiality:** IoT environments collect and transmit vast amounts of sensitive data, including personal, health, and financial information. Ensuring IDS-monitored data remain private and confidential is essential, given the critical nature of such environments. Furthermore, IDSs are vulnerable to adversarial attacks, leading it to fail to detect new threats.
- **High False Positive Rates:** Excessive false positives reduce the effectiveness of anomaly-based IDSs. This challenge arises from the difficulty in differentiating between benign anomalies and genuine attacks, resulting in excessive false alarms and diminished trust in the IDS. Incorporating advanced techniques, such as artificial intelligence (AI) and statistical models, can enhance accuracy and minimize false alerts.
- **Explainability:** Many IDS solutions, particularly those based on ML and DL, operate as “black boxes,” making decision-making processes opaque. A lack of transparency reduces trust and hinders adoption. XAI techniques can address this issue by understanding attack patterns and providing interpretations of detection results, which in turn supports and enhances the decision-making process.
- **Scalability:** The growing IoT ecosystem generates massive data volumes, increasing device interconnectivity and expanding attack vectors. To prevent IDS overload, scalable solutions such as distributed architectures, edge computing, and cloud-based approaches must be implemented.
- **Computational Complexity:** Due to the nature of IoT systems, integrating machine learning approaches introduces computational challenges that require additional resources and careful optimization to maintain system efficiency.
- **Evaluation Metrics:** IoT systems often handle sensitive data (e.g, healthcare date). However, existing assessment measures are unable to evaluate how successfully IDS maintain a balance between effective security detection and privacy preservation.

## 8. ML-Based Security Solutions in the IoT: Addressing Security, Privacy, and Trust

The rapid adoption of the IoT has intensified concerns regarding security, privacy, and trust across various applications. To mitigate these challenges, advanced ML methodologies, including DL, EL, federated learning (FL), and transfer learning (TL), have been explored. This section examines the role of ML-based solutions in enhancing IoT security across four key domains: the industrial Internet of Things (IIoT), the IoV, healthcare, and smart cities. By analyzing existing methodologies and identifying research gaps, this discussion underscores the potential of ML in mitigating threats and fostering resilient IoT ecosystems.

### 8.1. Smart Cities

Machine learning-based security solutions play a crucial role in smart city applications by enhancing attack detection, privacy protection, and trustworthiness in AI-driven systems. Rashid et al. [186] propose an intrusion detection system (IDS) leveraging classifiers such as Logistic Regression (LR), Support Vector Machine (SVM), Decision Trees (DTs), Random Forest (RF), Artificial Neural Networks (ANNs), and K-Nearest Neighbors (KNN). Ensemble methods, particularly stacking, demonstrated superior detection performance.

For privacy, El et al. [187] assess risks in smart cities arising from ubiquitous connectivity, smart cards, cloud computing, and autonomous systems. The study compares privacy-preserving techniques such as anonymization, encryption, and access control, emphasizing the necessity of a stakeholder-inclusive approach to ensure compliance with regulations like the General Data Protection Regulation (GDPR) and the Health Insurance Portability and Accountability Act (HIPAA).

Trustworthy ML models are vital in cloud-based services for smart cities, as highlighted by Qolomany et al. [188]. Their heuristic approach minimizes communication overhead while enhancing model reliability by evaluating trustworthiness based on historical data and agreement levels within an ensemble. This method was validated through traffic flow prediction and predictive maintenance case studies.

Kabir et al. [189] advocate for explainable artificial intelligence (XAI) to address the transparency and security challenges in AI-driven smart city applications. The study shows that XAI enhances interpretability without significantly affecting model performance, ensuring a balance between accuracy and explainability.

For secure data transmission, Annadurai et al. [142] introduce a biometric authentication-based IDS incorporating Kernel-Based Principal Component Analysis (KPCA) and VGG-16 for classification. The system, coupled with the Deterministic Trust Transfer Protocol (DTTP), achieves 96% accuracy while ensuring data integrity and privacy.

Abdalzaher et al. [190] propose a benchmarking framework categorizing ML models for IoT security challenges. The study evaluates linear models such as LR and SVM alongside non-linear models like RF, AdaBoost (AB), and KNN, focusing on anomaly and intrusion detection effectiveness. Liloja et al. [152] address security in IoT-enabled smart cities by introducing a hybrid IDS combining Random Forest with a Restricted Boltzmann Network (RF-RBN). The model, trained on the GPRS, CIDDS001, and UNSW-NB15 datasets, achieved high accuracy, sensitivity, and specificity in detecting attacks.

A lossless data-hiding scheme for secure urban sensing is proposed by Abbasi et al. [191]. Their approach employs dynamic quadtree N-bit localization, ensuring efficient data transmission while preserving multimedia content integrity, with a Peak Signal-to-Noise Ratio (PSNR) of 52.23 dB. Federated learning (FL) combined with adversarial training is explored by Utomo et al. [192] to counter adversarial attacks in smart city applications. Federated Adversarial Training (FAT) enhances robustness, though slight accuracy degradation was observed under Projected Gradient Descent (PGD)-based attacks.

Zhang et al. [133] emphasize the need for integrating security, privacy, and trust in smart city infrastructures. The study eåxplores encryption, blockchain, and authentication mechanisms to enhance IoT security and ensure sustainable urban development. Alrayes et al. [86] propose the IDCPRO-DLM model, integrating the Chaotic Poor and Rich Optimization Algorithm (CPROA) with deep learning. The model achieves a maximum accuracy of 98.53% on the CICIDS2017 dataset, efficiently detecting attacks such as Distributed Denial of Service (DDoS), PortScan, Brute Force, and Botnet.

Table 3 presents a summary of AI-based security models for IoT applications in smart cities, while Table 4 provides a comparative analysis of their performance metrics in enhancing IoT security.

#### 8.1.1. Observations and Lessons Learned

Smart city security solutions leverage advanced ML and DL methods to improve intrusion detection, privacy protection, and trustworthiness. Key insights include:

- **Federated learning and ensemble methods** improve both accuracy and privacy yet are constrained by computation and communication overheads.
- **Trust modeling** is being integrated into ML pipelines using historical and consensus-based approaches, though trust remains loosely defined and inconsistently evaluated.
- **XAI** emerges as essential for public-facing smart services, providing transparency without heavily compromising performance.
- **Multi-layered defenses,** including biometric and blockchain-based methods, show strong potential but lack extensive real-world testing.

#### 8.1.2. Future Work

- **Lightweight FL and XAI models** suitable for edge devices in dense urban networks.
- **Standardized trust frameworks** to guide deployment and evaluation of trustworthy AI in smart city services.
- **Interdisciplinary integration** of privacy-preserving ML with legal regulations such as GDPR.
- **Real-world smart city testbeds** to validate ML models at scale in heterogeneous environments.

### 8.2. Healthcare

Li et al. [193] propose ADDETECTOR, a privacy-preserving smart healthcare platform for early Alzheimer’s disease (AD) detection using FL and DP. ADDETECTOR utilizes IoT devices to collect and analyze audio data, extracting acoustic and linguistic features. It employs a three-tier architecture—user, client, and cloud layers—to ensure privacy and security. FL minimizes raw data transmission, while DP safeguards data aggregation integrity. Experimental results show an accuracy of 81.88%, demonstrating its viability for intelligent healthcare.

Iwendi et al. [194] introduce a Security of Things (SoT)-based intrusion detection system (IDS) for smart healthcare, leveraging Random Forest (RF) and Genetic Algorithms (GAs) for feature optimization. The system detects threats such as malware, unauthorized access, and denial-of-service (DoS) attacks, achieving a 98.81% detection rate and a 0.8% false alarm rate using the NSL-KDD dataset. The study underscores the significance of GA-based feature selection in optimizing system performance.

Siniosoglou et al. [128] propose a federated learning-based IDS for next-generation IoT (NG-IoT) healthcare, utilizing Generative Adversarial Networks (GANs) within a multi-layer federated framework. The system detects cyberattacks in Medical Cyber–Physical Systems (MCPS) while preserving patient privacy and minimizing communication overhead. GANs identify anomalies in patient records and network traffic data. Evaluations using public datasets show superior intrusion detection performance compared to centralized methods. The federated approach enhances security against threats like data modification, injection, and DoS attacks.

Otoum et al. [195] present a federated transfer learning-based IDS to secure the Internet of Medical Things (IoMT). The system employs a Deep Neural Network (DNN) to develop a global model from decentralized edge models, preserving data privacy. Transfer learning enables knowledge sharing without exposing sensitive data. Using the CICIDS2017 dataset, the IDS demonstrates improved accuracy, detection rate, and training efficiency over centralized learning approaches.

Hussain et al. [196] propose a machine learning framework for detecting malicious traffic in IoT healthcare environments. Their tool, IoT-Flock, generates real-time traffic from both legitimate and malicious IoT devices. The resulting dataset trains various ML classifiers for cybersecurity threat detection, particularly in critical applications like Intensive Care Units (ICUs), where breaches pose severe risks. Experiments with six ML classifiers validate the framework’s practical applicability in securing healthcare IoT systems.

The study conducted by [134] examines the security challenges inherent in healthcare systems that utilize big data and the Internet of Medical Things (IoMT). While the IoMT and edge computing facilitate remote monitoring and data-driven decision-making, they also introduce significant privacy and security vulnerabilities. This research underscores the necessity of implementing robust security measures in healthcare big data platforms, emphasizing the role of ML in mitigating these risks through privacy-preserving methodologies and intrusion detection systems (IDSs).

The work presented in [197] proposes a secure Internet of Things (IoT) healthcare architecture incorporating deep learning-based access management to safeguard medical data. The system integrates data isolation, encryption, and real-time analytics utilizing a Convolutional Neural Network (CNN) to differentiate sensitive health information from general data. Furthermore, a federated deep learning (FDL) model is employed to enhance access control by dynamically analyzing user characteristics and trust levels. The proposed model demonstrates an accuracy of 98% in access control, exhibiting robust performance across various access conditions.

The study by [198] introduces a deep learning-based approach aimed at securing IoT healthcare systems. The authors employ a CNN in conjunction with a Camel-based rotating panel signature to ensure secure data access and management. This methodology prioritizes patient privacy, maintains data integrity, and prevents unauthorized access within intelligent healthcare environments. Additionally, a cloud-based architecture is utilized for the secure storage and processing of sensitive patient data, while real-time monitoring facilitates continuous health tracking. The study highlights the necessity of stringent security protocols due to the inherent vulnerabilities associated with wireless networks and interconnected devices.

The research in [199] presents an AI-driven cybersecurity system tailored for healthcare applications, employing Multi-Source Transfer Learning (MSTL) within an Edge of Things (EoT) framework to detect and classify cyberattacks, including denial-of-service (DoS), distributed denial-of-service (DDoS), malware, and man-in-the-middle attacks. By integrating edge and cloud computing, the proposed approach ensures secure and efficient data transmission and processing. Experimental evaluations on datasets such as EMNIST, X-IIoTID, and Federated TON_IoT demonstrate significant improvements in cyber threat detection accuracy and execution time relative to existing methodologies.

To enhance cybersecurity within IoMT environments, ref. [153] introduces a secure ensemble learning methodology that employs a fog–cloud architecture integrating deep learning and ML techniques for cyberattack detection. The proposed system leverages Long Short-Term Memory (LSTM) networks as base learners and a Decision Tree (DT) for event classification. Evaluated on the ToN-IoT dataset, this methodology achieves superior accuracy, precision, and detection rates in comparison to conventional techniques.

The study in [147] proposes a privacy-preserving federated learning (FL) mechanism specifically designed for healthcare applications, enabling multiple institutions to collaboratively train ML models without exposing sensitive patient data. The proposed approach integrates secure multi-party computation (SMPC) and DP to ensure data security and model integrity. Experimental evaluations conducted on MIMIC-III and Synthea™ datasets illustrate promising results in both privacy preservation and model accuracy, demonstrating superior performance over traditional centralized ML methodologies.

Given the increasing reliance on IoMT and AI-driven technologies, securing medical data from cyberattacks such as malware, unauthorized access, and DoS remains a critical challenge. The study in [200] presents an ensemble-based intrusion detection system for healthcare (EIDS-HS), utilizing Support Vector Machine, Decision Tree, and K-Nearest Neighbors to effectively detect cyber threats. Evaluations conducted using the NSL-KDD dataset reveal that the proposed system achieves superior accuracy, recall, and F1-score compared to conventional intrusion detection methods. Additionally, formal security verification utilizing the Scyther tool confirms the model’s robustness against a range of cyber threats.

In [201], the authors propose a machine learning-based intrusion detection system (IDS) optimized using the metaheuristic Firefly Algorithm (FA). The study underscores the critical need for robust security mechanisms in IoT, particularly within the framework of Healthcare 4.0. The proposed IDS employs Extreme Gradient Boosting (XGBoost) as its core classification model, enhanced by a modified FA to improve the detection of cyber threats targeting healthcare IoT devices. Empirical evaluations demonstrate that the proposed approach achieves superior accuracy and precision compared to conventional machine learning models. Furthermore, SHapley Additive exPlanations (SHAP) analysis is utilized to enhance interpretability by identifying key features influencing model predictions.

The escalating cybersecurity challenges in the Internet of Medical Things (IoMT) have necessitated advancements in IDS methodologies, as examined in [202]. This study explores the application of ensemble learning techniques, including stacking and bagging, to enhance threat detection in IoMT environments. A significant contribution of this work is the suggestion of a performance-driven, weighted meta-learning framework, which dynamically assigns voting weights to classifiers based on evaluation metrics such as accuracy, loss, and confidence. By iteratively refining ensemble models to counter emerging threats, the proposed meta-learning-based IDS demonstrates superior performance relative to traditional models, particularly with respect to accuracy, detection rate, and false positive rate, thereby substantiating its efficacy in strengthening IoMT security.

Table 5 presents a taxonomy of AI-driven solution models for IoT healthcare applications, while Table 6 provides a comprehensive comparative analysis of the performance metrics of various AI algorithms, highlighting their contributions to enhancing IoT security within healthcare ecosystems.

#### 8.2.1. Observations and Lessons Learned

ML and DL techniques in Healthcare IoT enhance intrusion detection and privacy but face domain-specific constraints:

- **Federated learning** supports privacy-preserving training but introduces synchronization and convergence issues in resource-limited devices.
- **Ensemble and meta-learning** significantly improve detection accuracy and adaptiveness, particularly in dynamic health data environments.
- **Optimization methods** (e.g., firefly algorithm, genetic algorithms) refine model selection and reduce false alarms yet raise complexity and deployment overhead.
- **Interpretability** is crucial, especially when decisions affect patient care; however, few studies balance this with high performance.

#### 8.2.2. Future Work

- **Efficient FL systems** tailored to IoMT with reduced bandwidth and latency requirements.
- **Adaptive models for evolving threats**, capable of handling new attack patterns in real time.
- **Transparent decision-making tools** for clinical environments, combining interpretability with high detection accuracy.
- **Scalable healthcare IoT frameworks** tested on diverse clinical datasets and integrated with healthcare standards (e.g., HIPAA).

### 8.3. Internet of Vehicles (IoV)

Sharma et al. [154] propose a data-centric misbehavior detection model for IoV using machine learning techniques. The model analyzes inter-vehicle data to detect and classify misbehavior, such as position forgery, in vehicular networks. By integrating plausibility checks with six supervised machine learning algorithms, it enhances misbehavior detection accuracy and reliability. Performance evaluation with the VeReMi dataset, which simulates various vehicular misbehavior attacks, demonstrates the model’s effectiveness in identifying hazardous behaviors. This approach addresses the dynamic nature of IoV environments, ensuring real-time protection of Vehicle-to-Vehicle (V2V) communication with high precision and recall.

Yang et al. [203] develop a Multitiered Hybrid Intrusion Detection System (MTH-IDS) to enhance IoV security. The system protects both intravehicle networks (IVNs) and external vehicular networks by detecting known and zero-day threats through a hybrid intrusion detection system (IDS) that combines signature-based and anomaly-based mechanisms. Using tree-based supervised learning models for known attacks and k-means clustering for unknown (zero-day) attacks, MTH-IDS achieves detection rates of 99.99% on the CAN-intrusion dataset (IVN data) and 99.88% on the CICIDS2017 dataset (external network data).

Kumar et al. [204] introduce the Privacy-Preservation-Based Secured Framework for IoV (P2SF-IoV), integrating blockchain and deep learning to enhance privacy and security. The framework addresses data integrity, verifiability, scalability, and security vulnerabilities such as data poisoning and man-in-the-middle (MitM) attacks. Blockchain ensures secure data transfer and authentication among IoV nodes, Roadside Units (RSUs), and cloud servers, while Long Short-Term Memory (LSTM) networks perform intrusion detection. Validation with the IoT-Botnet and ToN-IoT datasets demonstrates superior privacy, detection accuracy, and scalability.

To detect data falsification attacks while preserving privacy, Uprety et al. [205] propose a federated learning (FL)-based Privacy-Preserving Misbehavior Detection System for Vehicular Ad-hoc Networks (VANETs). This system enables vehicles to train models locally using their own data, eliminating the need to share sensitive information with central authorities. A federated learning approach aggregates locally trained models on Basic Safety Message (BSM) data at a central authority, facilitating accurate detection of position falsification attacks while ensuring robust privacy protection.

In [135], the authors propose a framework to enhance privacy, efficiency, and scalability in service deployment for Internet of Vehicles (IoV) networks. This approach integrates deep reinforcement learning (DRL), specifically the Deep Deterministic Policy Gradient (DDPG) algorithm, with federated learning for collaborative, privacy-preserving service deployment. Homomorphic encryption ensures secure integration of model weights while maintaining privacy. Services are dynamically deployed based on real-time system requirements, reducing the computational burden on individual edge servers (ESs) while preserving data privacy.

Hbaieb et al. [206] propose a Software-Defined Networking (SDN) framework for the IoV incorporating a federated learning-based intrusion detection system (IDS). This system enhances malicious activity detection, such as node infiltration and black hole attacks while preserving data privacy. By leveraging trust metrics—such as traffic flow and packet drop rate—the framework improves detection accuracy, identifying potentially malicious nodes. The system achieves a recall of 99.04% and a precision of 99.30%, surpassing conventional IDS approaches. This research addresses IoV security and privacy challenges through federated learning’s distributed model training.

In [143], a transfer learning and Optimized Convolutional Neural Network (CNN)-based IDS is proposed to protect IoV systems from cyberattacks targeting intra-vehicle and external vehicular networks. This model integrates transfer learning with hyperparameter tuning to enhance CNN-based detection frameworks, including VGG16, VGG19, Xception, Inception, and InceptionResNet. Additionally, Particle Swarm Optimization (PSO) optimizes hyperparameters, improving attack detection. Evaluated on the Car-Hacking and CICIDS2017 benchmark datasets, the system achieves detection rates and F1-scores exceeding 99.25%, demonstrating high accuracy in intrusion detection.

Xu et al. [148] address security, reliability, and scalability challenges in knowledge transfer among vehicles in IoV networks using transfer learning (TL). The proposed model enhances TL trustworthiness through a reputation-based selection of reliable vehicles and consortium blockchain for decentralized reputation management. An auction-based incentive mechanism encourages high-reputation vehicle participation, ensuring dependable model sharing. Evaluated in a simulated trading market, the system demonstrates substantial accuracy and reliability in securing TL transactions within 6G-enabled IoV environments.

Ullah et al. [129] propose HDL-IDS, a hybrid deep learning intrusion detection system (IDS) designed for accurate detection of both inter-vehicle and intra-vehicle network intrusions. The system integrates Long Short-Term Memory (LSTM) and Gated Recurrent Units (GRUs) to improve the detection of cyberattacks, including Distributed Denial of Service (DDoS), fuzzing, and spoofing. Evaluated on the CICIDS2017, CSE-CIC-IDS2018, and Car-Hacking datasets, the model achieved 99.5% accuracy for DDoS attacks and 99.9% for car-hacking-related attacks. This hybrid approach reduces training time and response latency, enabling real-time detection suitable for Internet of Vehicles (IoV) applications.

To secure both intra-vehicle and inter-vehicular communications, Otoum et al. [207] propose a transfer learning-driven IDS for IoV. This model employs multi-task transfer learning to enhance cyberattack detection while reducing training time and computational costs. Utilizing Deep Neural Networks (DNNs) and Convolutional Neural Networks (CNNs), the IDS is trained to recognize threats across vehicular networks. The transfer learning approach allows knowledge transfer from smaller datasets to larger ones, improving detection accuracy and minimizing fine-tuning time.

Rani et al. [208] introduce a federated learning (FL)-based Misbehavior Detection Model for the 5G-Enabled IoV to address security and privacy challenges. FL enables malicious behavior detection without sharing sensitive local data. The model employs Federated Distillation (FD) to reduce communication overhead while maintaining high detection accuracy across multiple datasets, including ISCXIDS2012, CIC-IDS2017, CSE-CIC-IDS2018, and Car-Hacking. By transmitting average logit values instead of full model parameters, FD improves FL efficiency in IoV by minimizing communication frequency. The model achieves 99.72% detection accuracy, with a precision of 99.70%, recall of 99.20%, and an F1-score of 99.26%.

Gou et al. [209] propose a multi-classification and tree-based ensemble network for an IDS to enhance cyberattack detection in both intra-vehicle networks (IVNs) and external vehicular networks. The system leverages a tree-based ensemble learning approach, combining the Synthetic Minority Over-Sampling Technique (SMOTE) and Random Under-Sampler to address class imbalance. The model features a deep-layer architecture integrating ML models, including XGBoost, Random Forest (RF), and LightGBM. Evaluated on the CICIDS2017 and Car-Hacking datasets, it achieved an F1-score of 0.965 on CICIDS2017 and 0.9999 on the Car-Hacking dataset.

Wang et al. [210] introduce MESMERIC, a machine learning-based trust management mechanism for securing Internet of Vehicles (IoV) networks. It integrates direct and indirect trust while considering interaction context to detect and exclude malicious vehicles that threaten network integrity. MESMERIC employs machine learning to define an optimal decision boundary, distinguishing trustworthy from untrustworthy vehicles based on trust metrics such as interaction success rate, similarity, familiarity, and reward and punishment. Unlike traditional models that rely on static, human-assigned weights, MESMERIC reduces subjectivity by leveraging adaptive learning. Simulation results confirm its effectiveness in identifying malicious vehicles, outperforming conventional models. Table 7 presents AI-based models for IoT applications in the IoV, while Table 8 provides a comparative analysis of AI algorithms’ performance metrics in enhancing IoT security within IoV environments.

#### 8.3.1. Observations and Lessons Learned

IoV systems benefit from advanced ML models to address complex, fast-changing security demands:

- **Hybrid DL models** (e.g.,: LSTM and GRU) are highly effective at detecting network anomalies in real-time but consume significant resources.
- **Transfer learning** reduces training time and supports cross-domain knowledge reuse, improving attack detection with limited data.
- **Trust management systems**, especially those using contextual and adaptive metrics, enhance the resilience of vehicular networks.
- **Blockchain and federated learning** combinations improve privacy and integrity but often remain conceptual with limited field testing.

#### 8.3.2. Future Work

- **Edge-optimized IDS models** that balance detection precision with low latency and resource use.
- **Online learning systems** that continuously update with new threats in vehicular networks.
- **Blockchain-integrated FL** frameworks with minimal communication overhead.
- **Field-deployable prototypes** tested in diverse IoV environments (e.g.,: urban vs. highway) for performance and robustness.

### 8.4. Industrial Internet of Things (IIoT)

In [46], the authors propose a machine learning approach to detect false data injection (FDI) attacks in Industrial Internet of Things (IIoT) systems. The study utilizes Autoencoders (AEs) to capture temporal and spatial correlations in sensor data, mitigating the risk of FDI attacks, which manipulate sensor readings to mislead industrial processes. Denoising Autoencoders (DAEs) further refine data accuracy. Performance evaluations indicate the AE-based method surpasses traditional Support Vector Machine (SVM) techniques, achieving higher detection rates, fewer false alarms, and the capability to identify novel attacks without labeled data.

Latif et al. [55] introduce a Random Neural Network (RaNN) model for a lightweight intrusion detection system in IIoT environments. The study aims to enhance detection accuracy while minimizing prediction time, addressing cyber threats such as Denial of Service (DoS), data type probing, and malignant control. The RaNN model outperforms conventional methods, including Artificial Neural Networks (ANNs), SVM, and Decision Trees (DTs), in terms of accuracy, precision, recall, and F1-score. Trained on the DS2OS dataset, the model achieved 99.20% accuracy, demonstrating its efficacy in IIoT attack detection.

Hassan et al. [211] propose a deep learning-based trust boundary protection mechanism for IIoT environments to defend against adversarial attacks. The approach integrates a cooperative data generator with a downsampler-encoder architecture and a Deep Neural Network (DNN) discriminator. This method strengthens traditional ML models, which are often vulnerable to adversarial noise and dynamic attacks. Evaluated on real IIoT attack data, the system improves accuracy in detecting various threats, including Distributed Denial of Service (DDoS), command injection, and relay misconfiguration.

The PriModChain framework [212] introduces a privacy-preserving machine learning environment for IIoT security. Addressing privacy, security, and trust concerns in conventional ML systems, PriModChain integrates DP, federated learning (FedML), the Ethereum Blockchain (EthBC), and smart contracts to enhance privacy and security in distributed IIoT networks. The framework prevents adversarial attacks, data breaches, and privacy violations while enabling secure model training and sharing across distributed IIoT entities. This approach effectively classifies cyberattacks and safeguards sensitive IIoT data.

Taheri et al. [149] propose Fed-IIoT, a federated malware detection architecture designed for robustness in Industrial Internet of Things (IIoT) environments. It focuses on detecting Android-based malware using federated learning (FL) to enhance privacy by enabling collaborative model development without sharing raw data. Fed-IIoT integrates a generative adversarial network (GAN) to generate adversarial samples and mitigate poisoning attacks. Additionally, Byzantine defense mechanisms, such as Byzantine Median (BM) and Byzantine Krum (BK), protect against malicious actors. Evaluations on three IoT datasets show an 8% accuracy improvement over other malware detection methods.

Mothukuri et al. [80] develop a federated learning-based anomaly detection system for IoT networks, ensuring data privacy and security. The framework employs Gated Recurrent Units (GRUs) alongside FL to detect breaches while keeping data localized on IoT devices, sharing only model parameters. Tested on Modbus-based IoT network data, the system outperforms centralized approaches in accuracy and false alarm rate.

Ruzafa-Alc’azar et al. [213] introduce a privacy-preserving, FL-based intrusion detection system (IDS) for the IIoT. The system secures sensitive IIoT data while providing robust intrusion detection. Data remain on edge devices, with only model parameters shared. DP adds noise to model updates to prevent sensitive information leakage. Two aggregation techniques, FedAvg and the newly proposed Fed+, address non-independent and identically distributed (non-IID) data, a common IIoT challenge. The system effectively detects cyberattacks, including distributed denial-of-service (DDoS), backdoor, and command injection attacks, while maintaining data privacy and security.

To enhance anomaly detection in the IIoT, Wang et al. [136] propose a hierarchical FL framework integrating deep reinforcement learning (DRL). This scalable, privacy-preserving system mitigates security risks associated with decentralized data collection. It addresses the challenge of non-IID data, which affects centralized models. Experimental results demonstrate real-time anomaly detection in IIoT networks with minimal latency, high accuracy, and low false alarm rates while preserving data privacy.

TrustFed, a blockchain-based framework, ensures impartiality and trust in cross-device federated learning (CDFL) for Industrial Internet of Things (IIoT) environments [214]. Federated learning enables IIoT devices to collaboratively train machine learning models while preserving privacy. However, adversarial attacks, model poisoning, and unfair training practices pose threats to decentralized systems. By integrating blockchain technology, TrustFed ensures that only trusted devices contribute to model updates and maintains device reputations, addressing these challenges. It enhances fairness by filtering out outliers and detects malicious devices using Ethereum-based smart contracts. The framework demonstrates superior attack detection, model accuracy, and impartiality on an IIoT dataset compared to traditional approaches.

A hybrid deep learning framework is proposed in [144] to detect and mitigate botnet attacks in IIoT environments. The authors present a scalable botnet detection solution by combining Deep Neural Networks (DNNs) and Long Short-Term Memory (LSTM) models. This approach addresses IIoT security complexities, including real-time detection of multi-variant botnet attacks and the distributed nature of IIoT systems. The N_BaIoT dataset, containing malicious traffic from Gafgyt and Mirai botnets across multiple IoT devices, is used for evaluation. Results show that the hybrid LSTM-DNN model outperforms existing methods, achieving a 99.94% detection rate with a minimal processing time of 0.066 milliseconds.

In [215], a solution is proposed to address imbalanced multiclass data in IIoT intrusion detection systems (IDSs). The model leverages the XGBoost algorithm to improve detection accuracy for various intrusions in IIoT environments. XGBoost is chosen for its ability to handle imbalanced datasets, scale efficiently, and deliver high classification accuracy. Performance is evaluated using two benchmark IIoT datasets: X-IIoTID and TON_IoT, which present imbalanced challenges for conventional IDS systems. Results show that the XGBoost model significantly enhances detection accuracy for attack types such as ransomware, distributed denial-of-service (DDoS), and command injection attacks, achieving an F1-score of 99.9% on X-IIoTID and 99.87% on TON_IoT.

In [216], a deep learning-based IDS, DL-IDS, is proposed to protect IoT environments from various attacks. The framework integrates the Spider Monkey Optimization (SMO) algorithm with a Stacked-Deep Polynomial Network (SDPN) to enhance intrusion detection accuracy and efficiency. The SDPN classifies traffic as normal or anomalous, while the SMO algorithm selects optimal features, reducing dimensionality and improving classification speed. The NSL-KDD dataset, which includes attack types such as denial-of-service (DoS), remote-to-local (R2L), user-to-root (U2R), and probe attacks, is used for evaluation. Results indicate that DL-IDS outperforms conventional IDS models, achieving 99.02% accuracy, along with superior precision, recall, and F1-score values.

A stacked ensemble classifier for intrusion detection systems (IDSs) in edge-based Internet of Things (IoT) and industrial IoT (IIoT) networks is proposed in [130]. To enhance detection accuracy and reduce computational costs in distributed environments, the framework integrates multiple machine learning models, including the Bidirectional Gated Recurrent Unit–Recurrent Neural Network (B-GRU-RNN), Random Forest (RF), and Deep Neural Network (DNN). By leveraging the strengths of these models, the framework optimizes intrusion detection for threats such as distributed denial-of-service (DDoS), ransomware, and brute force attacks. Evaluated using the TON IoT dataset, which includes real-world IoT and IIoT device data, the ensemble classifier outperformed individual models, achieving an average accuracy of 99.7

In [217], a Data Fusion and Transfer Learning-Empowered Granular Trust Evaluation (DFTE) mechanism is introduced for IoT networks, emphasizing IIoT security. The system integrates deep reinforcement learning (DRL) with transfer learning (TL) to develop fine- and coarse-grained trust evaluation models. This hybrid approach assesses user trustworthiness in IIoT ecosystems by analyzing actions and task completions, ensuring data reliability. Transfer learning improves efficiency by reducing training time through knowledge reuse. The DFTE architecture incorporates a dynamic reward and punishment system to promote honest behavior and penalize malicious actions. Experimental results demonstrate high accuracy in trust evaluation, significantly enhancing user engagement and data reliability in IIoT environments.

In [49], a deep learning-based IDS is proposed to secure IIoT networks using various ML and DL models, including Bidirectional Long Short-Term Memory (Bi-LSTM), Gated Recurrent Units (GRUs), and Long Short-Term Memory (LSTM) for binary and multi-class classification tasks. The approach addresses issues such as outdated datasets, data imbalance, and limited attack detection by applying Singular Value Decomposition (SVD) for feature reduction and the Synthetic Minority Oversampling Technique (SMOTE) for data balancing. The model achieves 99.99% accuracy for binary classification and 99.98% for multi-class classification. It effectively detects multiple attack types, including backdoor, DDoS, injection, ransomware, and scanning. Table 9 presents AI-based solution models for IoT applications in the IIoT, while Table 10 provides a comparative analysis of various AI algorithms’ performance in enhancing IoT security in IIoT environments.

#### 8.4.1. Observations and Lessons Learned

ML-based solutions in the IIoT tackle diverse threats but face unique industrial constraints:

- **FL and blockchain-based architectures** preserve privacy and decentralize learning, essential for multi-stakeholder industrial systems.
- **Autoencoders and hybrid DL models** excel at anomaly and intrusion detection, especially for unknown or stealthy attacks.
- **Optimization techniques** like SMO and GANs enhance performance but demand careful tuning and add overhead.
- **Handling non-IID data** and imbalanced datasets is a recurring challenge, with transfer learning showing promise in addressing it.

#### 8.4.2. Future Work

- **Lightweight and scalable IDS frameworks** capable of real-time detection under constrained computation.
- **Federated anomaly detection** models resilient to adversarial and poisoning attacks in heterogeneous IIoT setups.
- **Explainable AI** for critical industrial systems to support traceable decision-making in high-risk environments.
- **Cross-domain generalization** strategies to ensure IDS adaptability to new attack vectors or evolving industrial protocols.

Table 11 presents a comprehensive summary of AI approaches applied in IoT security. It outlines their application domains, key strengths, current limitations, and suggested future research directions. While prior studies have focused on specific use cases and algorithms, this synthesis provides cross-domain insights to guide the development of scalable, efficient, and secure IoT systems. The categorization is based on the surveyed literature from 2020 to 2024, covering the smart city IoT, healthcare IoT, IoV, and IIoT.

## 9. Conclusions

The IoT represents a transformative technological paradigm that interconnects devices, facilitating seamless communication across various domains, including transportation, healthcare, industry, and urban management. Its architectural foundation comprises multiple layers designed to enable efficient data collection, processing, and user interaction. The global IoT market is projected to experience substantial growth in the coming years, underscoring its increasing significance.

Key applications of IoT, such as the IoV, healthcare IoT, IIoT, and smart city IoT, have introduced innovative solutions that enhance safety, efficiency, and convenience. However, the increasing reliance on IoT within these domains presents critical challenges, particularly concerning security, privacy, and trust. The IoV is vulnerable to risks associated with unauthorized access and data integrity breaches. The healthcare IoT necessitates stringent privacy measures due to the sensitivity of patient data. The IIoT requires robust protection mechanisms to safeguard industrial processes against cyber threats and disruptions. Meanwhile, the smart city IoT demands comprehensive frameworks to secure large-scale infrastructure while maintaining public trust.

Effectively addressing these challenges is crucial for the continued advancement and secure deployment of IoT applications. Future developments must integrate comprehensive security protocols, privacy-enhancing mechanisms, and trust management strategies to mitigate risks and ensure the reliable, efficient, and ethical utilization of interconnected systems across industries.

ML serves as a fundamental enabler of the IoT, augmenting security, optimizing resource allocation, and enabling intelligent decision-making through data-driven insights. This study examines various ML techniques, including supervised, unsupervised, and reinforcement learning, highlighting their role in IoT applications such as anomaly detection, predictive maintenance, and cybersecurity.

Supervised learning demonstrates significant efficacy in identifying patterns within labeled data, contributing to applications such as industrial equipment monitoring and smart home security. Conversely, unsupervised learning facilitates the discovery of hidden patterns within large datasets without labeled outputs, rendering it particularly useful for anomaly detection in IoT environments. Reinforcement learning further enhances IoT systems by enabling dynamic decision-making, thereby improving resource allocation and energy management.

Advanced ML techniques, such as deep learning, bolster IoT security by detecting anomalies and identifying cyber threats through neural network-based analysis. Ensemble learning enhances model accuracy by integrating multiple algorithms, while federated learning supports privacy-preserving model training across distributed IoT devices. Transfer learning enables the efficient adaptation of pre-trained models to IoT-specific security challenges, thereby reducing the necessity for extensive data collection.

Overall, ML empowers IoT systems to function with heightened efficiency and security in complex, data-intensive environments. By leveraging diverse ML techniques, the IoT continues to evolve into a more intelligent and responsive ecosystem, addressing challenges related to anomaly detection, security threats, and resource management.

The rapid proliferation of IoT technologies has significantly enhanced automation and connectivity across multiple sectors. However, this expansion has concurrently introduced critical security vulnerabilities, largely due to the widespread reliance on unencrypted communication, weak authentication mechanisms, and susceptibility to cyberattacks. This study underscores the imperative for robust security measures tailored to each IoT domain to safeguard data integrity, privacy, and operational reliability.

Fundamental security requirements include data integrity, access control, privacy protection, secure communication, firmware integrity, and regulatory compliance. Each IoT domain presents distinct security challenges that necessitate specialized defense mechanisms. This study categorizes major cyber threats, including DoS/DDoS attacks, data breaches, unauthorized access, poisoning attacks, and malware/botnets, illustrating their specific ramifications for various IoT applications.

Emerging cybersecurity strategies, such as federated learning, blockchain-based encryption, anomaly detection systems, and AI-driven security models, have demonstrated considerable potential in mitigating cyber threats. Nevertheless, the evolving nature of cyberattacks necessitates the continuous refinement of security frameworks, proactive threat detection, and industry-wide collaboration to ensure the effective protection of IoT ecosystems.

Securing IoT applications requires a multi-layered approach that integrates cryptographic techniques, ML-based detection systems, and adherence to regulatory compliance standards to protect critical infrastructure and sensitive data from cyber threats.

IDSs play a pivotal role in defending IoT environments against a broad spectrum of cyber threats. Traditional IDS methodologies, including signature-based and anomaly-based detection, have evolved to incorporate ML-driven and hybrid models, enhancing their accuracy and adaptability. However, the inherent complexity of IoT networks presents notable challenges, such as high false positive rates, scalability limitations, privacy concerns, and the demand for more interpretable AI-driven models. Future research should prioritize the development of adaptive, scalable, and transparent IDS solutions that leverage distributed architectures, edge computing, and explainable AI methodologies. As IoT ecosystems expand, ensuring the effective and efficient deployment of IDS remains critical to maintaining cybersecurity resilience.

The conclusions drawn from this study underscore the transformative role of ML-driven security mechanisms in mitigating evolving threats within IoT environments. This research highlights how ML methodologies, including deep learning, ensemble learning, federated learning, and transfer learning, significantly enhance IoT security across domains such as smart cities, healthcare, the IIoT, and the IoV.

Key findings indicate that ML-based security frameworks improve real-time threat detection, mitigate cyberattacks, and enhance privacy protection. However, persistent challenges remain, including high computational costs, scalability limitations, adversarial vulnerabilities, and the necessity for more interpretable ML models. Future research should focus on optimizing computational efficiency, integrating privacy-preserving techniques such as DP and blockchain and enhancing the trustworthiness of ML-driven security solutions. In particular, it is important to specify the use of noise addition strategies in DP, such as Laplace or Gaussian mechanisms, to define clear privacy guarantees. Additionally, the design of smart contracts should support access control, privacy budget management, and auditability to ensure secure and transparent operation in decentralized systems.

This study synthesizes findings from a wide range of published research to compare the performance of ML models for IoT security across different domains. While detailed performance metrics—such as accuracy, false positive rate, and computational overhead—are summarized from the literature, no new empirical benchmarking was conducted. We acknowledge this as a limitation and recommend that future work implement unified experimental comparisons using standardized datasets to validate and contextualize the comparative insights presented in this study.

Furthermore, this study emphasizes the need for scalable and adaptive security solutions capable of addressing the dynamic nature of IoT threats. Emerging technologies, including edge computing, federated learning, and explainable AI, represent promising avenues for strengthening security and ensuring compliance with evolving regulatory frameworks.

Ultimately, securing IoT ecosystems necessitates continuous innovation, interdisciplinary collaboration, and real-world validation of proposed security models. By addressing these challenges and leveraging advanced ML techniques, IoT security can be substantially reinforced, fostering resilient, privacy-conscious, and trustworthy infrastructures.

While this study offers a broad and integrative perspective across various IoT domains, we acknowledge that such comprehensive coverage necessarily limits the depth of analysis within each individual application area. This approach was intended to provide a unified taxonomy and cross-sectoral understanding of ML-driven security strategies, thereby laying the groundwork for more focused future research. Subsequent studies could delve deeper into domain-specific issues, such as adversarial robustness in healthcare IoT or real-time anomaly detection in IIoT, building on the insights presented in this study.

## Figures and Tables

**Table 1:** Comparison of our survey with recent surveys on IOT in terms of security, trust, and privacy using AI approaches.

Citation	Year	Security	Trust	Privacy	AI Approaches					IoT Applications				Comparison
					ML	DL	EL	TL	FL	Smart City	IoV	IIoT	Health- care	
[17]	2020	Yes	No	Yes	Yes	Yes	Yes	No	No	Yes	Yes	Yes	Yes	Focused on ML/DL for IoT security across smart cities, IoV, IIoT, and healthcare but lacks trust mechanisms, TL, and FL.
[18]	2020	Yes	No	Yes	Yes	Yes	No	No	No	No	Yes	No	No	Emphasizes ML/DL for IoT intrusion detection, lacks application to other IoT applications.
[14]	2020	Yes	No	Yes	Yes	Yes	No	No	Yes	No	No	No	No	General IoT security with ML/DL, lacks application of EL, TL.
[19]	2021	Yes	No	Yes	Yes	Yes	No	No	No	No	No	No	Yes	Focus on ML in healthcare IoT, lacks integration with broader IoT applications.
[20]	2021	Yes	Yes	Yes	Yes	Yes	Yes	No	No	Yes	No	Yes	Yes	Focuses on ML/DL for IIoT security, with limited coverage of other IoT applications.
[13]	2021	Yes	Yes	Yes	No	No	No	No	No	No	No	No	Yes	Focus on healthcare IoT security and standards, lacks ML techniques like EL, TL, FL.
[21]	2021	Yes	No	Yes	Yes	Yes	No	No	No	No	No	No	Yes	Focus on AI/ML for healthcare IoT security, lacks exploration of broader IoT applications.
[22]	2021	Yes	Yes	No	Yes	Yes	No	No	No	No	Yes	No	No	Focus on IoV, highlights ML/DL, lacks EL, TL, FL and other IoT applications.
[23]	2024	Yes	Yes	Yes	Yes	Yes	No	No	No	No	Yes	No	No	Focus on IoV security and trust, limited ML techniques such as DL, TL and FL.
[24]	2022	Yes	Yes	Yes	No	No	No	No	No	Yes	No	No	No	Focus on smart city security and privacy, but lacks exploration of ML techniques.
[25]	2022	Yes	No	Yes	Yes	Yes	No	No	No	No	No	No	No	Focus on ML/DL for intrusion detection, general IoT security but lacks broader IoT security applications.
[12]	2020	Yes	Yes	Yes	Yes	Yes	No	No	No	Yes	No	No	No	General IoT security with ML/DL, lacks detailed analysis of specific IoT applications.
[26]	2022	Yes	Yes	Yes	No	No	No	No	No	Yes	No	No	No	Focus on smart cities, lacks advanced ML techniques and broader IoT applications.
[7]	2023	Yes	Yes	Yes	Yes	Yes	No	No	No	Yes	No	No	No	Comprehensive review of smart city networks, lacks in-depth analysis of advanced ML techniques.
[8]	2023	Yes	Yes	Yes	Yes	Yes	No	No	Yes	No	No	Yes	No	Focus on IoT security using FL and DL, limited to IoT applications, lacks broader ML techniques.
[27]	2023	Yes	No	Yes	Yes	Yes	No	No	No	No	No	No	Yes	Focus on ML for IoT healthcare security, lacks exploration of other IoT applications.
[28]	2023	Yes	No	Yes	Yes	Yes	No	No	No	No	No	No	Yes	Focus on H-IoT security, highlights ML/DL, lacks broader IoT applications.
[29]	2023	Yes	Yes	Yes	Yes	Yes	No	No	Yes	Yes	No	No	No	Focus on FL for smart cities, lacks exploration of ML techniques across other IoT applications.
Our survey	2024	Yes	Yes	Yes	Yes	Yes	Yes	Yes	Yes	Yes	Yes	Yes	Yes	Comprehensive focus on security, trust, and privacy using all advanced ML techniques (ML, DL, EL, TL, FL) across smart cities, IoV, IIoT, and healthcare applications.

**Table 2:** Summary of intrusion detection techniques.

Citation	Year	Techniques	Type of Threats	Dataset	Accuracy
[160]	2020	Anomaly detection	Different types of threats	NSL-KDD dataset	98%
[161]	2020	A hybrid of anomaly detection and DL	DDoS, DoS, Scan, and Theft	Bot-IoT dataset	Not mentioned
[162]	2022	Anomaly detection and CNN	Abnormal traffic behavior	NID and BoT-IoT	99.51% and 92.85%
[163]	2019	A hybrid of anomaly detection and DL	Blackhole, Opportunistic Service, DDoS, Sinkhole, and Wormhole	5 million network transactions	97%
[164]	2019	A hybrid of signature-based IDS and anomaly-based IDS	DoS	Ips datasets	Not mentioned
[165]	2021	ANN	DoS, Probe, U2R, and R2L	KDD99	Not mentioned
[166]	2023	A hybrid of anomaly detection and PCC-CNN	Abnormal traffic behavior	NSL-KDD, CICIDS-2017	Above 98%
[167]	2024	Anomaly detection with KELM	Different types of threats	Not mentioned	99.40%
[168]	2018	Signature-based IDS	Abnormal traffic behavior	NSL-KDD	Not mentioned
[169]	2018	Signature-based IDS	DoS	-	Not mentioned
[170]	2022	Signature-based IDS	Malicious Intrusions	-	98.9%
[171]	2021	A hybrid of signature-based IDS and anomaly-based IDS	Different types of threats	NSL-KDD	Not mentioned
[172]	2022	A hybrid of signature-based and behavior-based detection	Anomalous behaviors caused by malicious activity	SWaT dataset	Above 96.0%
[173]	2024	A signature-based wireless intrusion detection system	Man-in-the-middle attacks	AWID3 dataset	True positive rate of 90%
[174]	2024	A distributed and cooperative signature-based intrusion	Man-in-the-middle attacks	AWID3 dataset	98%
[175]	2021	LR, SVM, RF	Botnet	UCI’s machine learning repository	Above 99%
[176]	2024	Anomaly detection and DT, RF, KNN, SVN	Denial of service attack	IoTID20 dataset	Above 99%
[177]	2020	RF, AdaBoost, GB, extremely randomized trees, classification RT, MLP	Denial of service attack	CIDDS-001, UNSW-NB15, and NSL-KDD	96.74%
[180]	2024	DL	Different types of threats	NSL-KDD and BoT-IoT	99.95%
[181]	2023	DL	Malicious Intrusions	NSL-KDD, KDDCup99, and UNSW-NB15	97%
[178]	2022	SVM, RF, KNN, DT, CNN	Different types of threats	CICIDS2017 dataset	Above 99%
[179]	2021	Honeypot with ML	Botnet	Botnet dataset	96%

**Table 3:** AI-Based Solution Models for IoT Applications in Smart Cities.

Citation	Year	IoT App	Focus Area	Ai Approche	Security Application	Advantages	Limitations
[186]	2020	Smart City	Security	ML and EL	IDS	High accuracy, precision, recall; Ensemble methods improve detection	High computational cost in ensemble models
[187]	2020	Smart City	Privacy	ML	Secure Data Communication	Holistic approach, integration of privacy laws	Limited testing in real-world environments
[188]	2020	Smart City	Trust	ML	traffic flow prediction	Reduced cloud overhead, high trust level (0.7–2.53% drop)	Not suitable for federated learning; limited to poisoning attacks
[189]	2022	Smart City	Security	ML	Cybersecurity enhancement	Improves transparency and trust in AI decisions	Trade-off between accuracy and explainability
[142]	2022	Smart City	Security	DL and TL	Intrusion Detection	High accuracy (96%), robust data transmission	High computational demand for real-time systems
[190]	2022	Smart City	Security	ML	IDS, Anomaly Detection	High accuracy, Robust against noisy data	High computational cost for large-scale systems
[152]	2023	Smart City	Security	ML and DL	IDS	High accuracy (95%), Addresses data imbalance	High computational complexity, particularly in feature extraction and model training stages, which may hinder real-time deployment in large-scale IoT networks
[191]	2023	Smart City	Security	ML	Lossless secure communication for IoT networks	High embedding capacity, low computational complexity	Limited testing on real-world large-scale systems
[192]	2023	Smart City	Privacy/ Security	FL	Intrusion Detection	Enhances privacy and security, robust against PGD and FGSM	Reduced accuracy with PGD-based attacks (10% drop)
[133]	2023	Smart City	Security/ Privacy	ML	Secure Data Communication	Promotes trust, privacy, and security in smart cities	Implementation challenges in large-scale systems
[86]	2023	Smart City	Security	DL	IDS	High accuracy (98.53%), improved feature selection	Computationally intensive for large-scale systems

**Table 4:** Performance of different AI algorithms for IoT security in smart cities.

Ref	Model	Attack Types	Performance Metrics Used	Results
[186]	Stacking	Various IoT cyberattacks	Accuracy, Precision, Recall, F1-Score	Stacking achieved highest performance with F1-score of 99.9%
[187]	ML	Data breaches, unauthorized access	Privacy, Compliance	Enhanced privacy, compliance with GDPR
[188]	Heuristic ML Model Selection	Poisoning attacks	Trust Level, Accuracy	Trust level decreased by 0.49–3.17% compared to ILP
[189]	XAI	General cyber threats, vulnerabilities	Accuracy, Transparency, Trust	High interpretability with moderate impact on accuracy
[142]	KPCA with VGG-16 Net and DTTP	Biometric-based intrusion detection	Accuracy, Precision, Recall, F-Score, RMSE	96% accuracy, improved recall 80%, RMSE of 46%
[190]	Random Forest, KNN, AdaBoost	Intrusion detection, anomalies	Accuracy, Precision, Recall, F1-Score	Achieved 95% accuracy with high precision
[152]	RF-RBN	Multiple IoT attacks (e.g., DoS, Spoofing)	Accuracy, Sensitivity, Specificity	95% accuracy, 96% sensitivity, 97% specificity
[191]	Quadtree N-bit localization-based RDH	Passive attacks, replay attack	PSNR, SSIM, Embedding Capacity	High PSNR (52.23 dB), high SSIM, large embedding capacity
[192]	FAT	PGD, FGSM	Accuracy, Robustness	81.13% (PGD) and 91.34% (FGSM) accuracy
[133]	privacy and security framework	Cyberattacks, data breaches	Data Privacy, Trust, Security	Enhanced data privacy and security with blockchain technology
[86]	IDCPRO-DLM (CPROA with DSAE)	DDoS, PortScan, Brute Force, Botnet	Accuracy, Precision, Recall, F1-Score	98.53% accuracy, high precision and recall

**Table 5:** AI-based solution models for IoT applications in healthcare.

Citation	Year	Focus Area	IoT Apps.	AI Approches	Security Application	Advantages	Limitations
[193]	2021	Privacy	Healthcare	FL	Data Protection, Privacy Preservation	High accuracy, privacy-preserving architecture	Decreased accuracy with high privacy settings
[194]	2021	Security	Healthcare	ML	IDS	High detection rate, low false alarm rate, optimized feature selection	Computationally intensive in large-scale environments
[128]	2021	Security, Privacy	Healthcare	FL	IDS	Improved privacy, decentralized model, higher detection accuracy	Computational overhead, complexity in large-scale systems
[195]	2021	Privacy, Security	Healthcare	FL, TL, DL	IDS	High accuracy, data privacy preserved, low communication overhead	High computational demand in federated settings
[196]	2021	Security	Healthcare	ML	IDS	Real-time traffic generation, high detection accuracy	High computational complexity for large-scale networks
[134]	2022	Security, Privacy	Healthcare	ML	Intrusion Detection, Data Protection, Privacy Preservation	Real-time monitoring, high accuracy in threat detection	Complexity in handling big data systems, scalability issues
[197]	2022	Privacy, Security	Healthcare	DL, FL	Data Protection, Privacy Preservation, Access Control	High accuracy, low privacy leakage, scalable for large datasets	High computational cost, requires frequent updates
[198]	2022	Security, Privacy	Healthcare	DL	Data Protection, Access Control, Privacy Preservation	High privacy, secure real-time monitoring, scalable	High computational complexity in resource-constrained environments
[199]	2023	Security	Healthcare	TL	Cyber Attack Detection, Malware Detection,	High accuracy, real-time processing, low latency	High computational demand in complex systems
[153]	2023	Security	Healthcare	ML, DL	IDS	Improved detection rate, real-time processing, efficient for IoMT	High computational overhead in fog nodes
[147]	2023	Privacy	Healthcare	FL	Data Protection, Privacy Preservation	Strong privacy guarantees, decentralized learning	High computational complexity in large-scale systems
[200]	2023	Security	Healthcare	ML, EL	IDS	High accuracy, formal security verification	Computational overhead with large datasets
[201]	2023	Security	Healthcare	ML	IDS	Improved accuracy, optimized model using FA, interpretable with SHAP	Computationally intensive for larger IoT systems
[202]	2024	Security, Privacy	Healthcare	ML, EL	IDS	High accuracy, adaptive, low false positive rate	High computational cost in large-scale IoMT environments

**Table 6:** Performance of different AI algorithms for IoT security in healthcare.

Ref	Model	Attacks Types	Performance Metric Used	Results
[193]	FL	Data leakage, MITM, dackdoor attack, data tampering	Accuracy, Execution Time	81.88% Accuracy, 0.712s detection time per user
[194]	GA-, RF	DoS, Probe, R2L, U2R	Detection Rate, False Alarm Rate, F1-Score	Detection Rate: 98.81%, False Alarm Rate: 0.8%, F1-Score: optimized by 8.2%
[128]	Federated GAN	Data modification, DoS, eavesdropping, MITM, data injection	Accuracy, F1-Score, Detection Rate	Accuracy: 92.98%, F1-Score: 0.928, Detection Rate: 91.5%
[195]	FTL with DNN	Data modification, DoS, data injection, unauthorized access	Accuracy, Detection Rate, Training Time	Accuracy: 95.14%, Detection Rate: 94.74%, Training Time: Reduced by 12.5%
[196]	RF, KNN, DT, LR	MITM, DDoS, spoofing, replay attacks	Accuracy, F1-Score, Precision, Recall	Random Forest achieved 99.51% Accuracy, F1-Score of 99.65%
[134]	ML	Data breaches, privacy violations, MITM, insider threats	Detection Rate, Accuracy, Privacy Preservation	High Detection Accuracy (>95%), strong privacy guarantees
[197]	FDL with CNN	Unauthorized access, rivacy leakage, data breaches	Accuracy, Precision, F1-Score	Accuracy: 98%, Precision: 95%, F1-Score: 0.95
[198]	CNN	Unauthorized access, data tampering	Accuracy, Security Level, Processing Time	Accuracy: 98%, low latency, improved security of patient data
[199]	CMTL	DoS/DDoS attacks, malware, injection, MITM,	Accuracy, Execution Time, F1-Score	Achieved high Accuracy (up to 99.24%) for 2 classes and improved execution time compared to other models
[153]	LSTM, DT	Data breaches, DoS/DDoS attacks, malware, injection, ransomware	Accuracy, F1-Score, Detection Rate, False Alarm Rate	Accuracy: 98.5%, F1-Score: 0.96, False Alarm Rate: 2.1%, Detection Rate: 97.8%
[147]	FL with DP and SMPC	Data leakage, MITM, backdoor, data tampering	Accuracy, Privacy Leakage, Computation Time	Accuracy: 97.69%, Privacy Leakage: 0.025, computation time improved by 15%
[200]	SVM, DT, KNN	Malware injection, replaying of information, unauthorized healthcare data disclosure, impersonation, credential guessing, DoS	Accuracy, F1-Score, Detection Rate	Accuracy: 95.12%, F1-Score: 0.94, Detection Rate: 94.74%
[201]	XGBoost	Data leakage, model poisoning, data tampering	Accuracy, Precision, Recall, F1-Score	Accuracy: 99.51%, Precision: 99.65%, Recall: 99.42%, F1-Score: 99.53%
[202]	Meta-Learning-	Data breaches, malware, DoS attacks, unauthorized access	Accuracy, F1-Score, Detection Rate, False Positive Rate	Accuracy: 98.0%, F1-Score: 0.996, Detection Rate: 97%, False Positive Rate: 0.101

**Table 7:** AI-based solution models for IoT applications in IoV.

Citation	Year	IoT Apps	Focused Area	Ai Approches	Security Application	Advantages	Limitations
[154]	2020	IoV	Security, Trust	ML and FL	Misbehavior Detection	High detection accuracy, enhanced with plausibility checks	Poor performance in detecting subtle position forgeries, computational complexity in large-scale environments
[203]	2021	IoV	Security, Trust	ML	Intrusion Detection	High accuracy, detection of zero-day attacks, real-time applicability	Computational complexity, struggles with random attack detection
[204]	2021	IoV	Privacy, Security, Trust	DL	Intrusion Detection	High privacy and security, scalability via IPFS	Computational overhead due to LSTM-based IDS
[205]	2021	IoV	Privacy, Security	FL	Misbehavior Detection	Privacy preservation, high detection accuracy, low communication overhead	Scalability issues, possible training errors due to wireless resource limitations
[135]	2022	IoV	Privacy, Security	DL and FL	Privacy-preserving service deployment	High coverage, low latency, privacy preservation	High computational complexity, high communication overhead
[206]	2022	IoV	Security, Trust, Privacy	FL	Intrusion Detection	High detection accuracy, privacy-preserving	High communication overhead, dependency on stable SDN
[143]	2022	IoV	Security	TL and EL	Intrusion Detection	High accuracy, optimized through PSO, scalable	High computational cost, limited real-time capabilities
[148]	2022	IoV	Security, Trust, Privacy	TL	Knowledge Transfer	Secure and reliable model sharing, high scalability	High computational cost due to auction-based model sharing
[129]	2022	IoV	Security, Privacy, Trust	DL	Intrusion Detection	High detection accuracy, reduced training time, real-time detection	Computational overhead due to deep learning complexity
[207]	2022	IoV	Security, Privacy	TL and DL	Intrusion Detection	High accuracy, reduced training time, knowledge transfer	High computational cost for deep learning models
[208]	2023	IoV	Security, Privacy	FL	Misbehavior Detection	High accuracy, low communication overhead	Limited scalability in highly dynamic environments
[209]	2023	IoV	Security, Privacy, Trust	ML	Intrusion Detection	High detection accuracy, addresses class imbalance	Computational overhead due to deep-layer ensemble learning
[210]	2024	IoV	Security, Trust	ML	Trust Management	Accurate trust segregation, context-aware, ML optimization	High computational complexity due to model training

**Table 8:** Performance of different AI algorithms for IoT security in IoV.

Ref	Model	Attacks Types	Performance Metric Used	Results
[154]	Supervised ML	Position Forgery	Precision, Recall, F1-score	5% improvement in precision, 2% in recall
[203]	Supervised ML	DoS, Fuzzy, Spoofing, Zero-Day	Accuracy, F1-score	99.99% (IVNs), 99.88% external
[204]	LSTM	DoS, Data Poisoning, MitM, Spoofing	Detection Rate, Accuracy	Over 99% accuracy, 0.00002–0.00451% FAR
[205]	FL with ANN	Position Falsification	Precision, Recall, Accuracy	Federated training outperformed central training in precision and recall
[135]	DRL, DDPG, FL	Privacy Leakage, Data Breaches	Coverage rate, delay, data transmission	82.6% coverage, reduced execution delay, reduced data transmission
[206]	FL	Black Hole, Malicious Node Infiltration	Recall, Precision, F1-score	99.04% recall, 99.3% precision
[143]	TL with CNN	DoS, Fuzzy, Spoofing, RPM Attacks	Accuracy, Precision, F1-score	Over 99.25% detection rate
[148]	TL	Data Poisoning, Model Tampering, Malicious Sellers	Detection Rate, Accuracy	Improved model accuracy, reduced adversarial effects
[129]	LSTM and GRU	DDoS, Fuzzy, Spoofing	Accuracy, Precision, F1-score	99.5% accuracy
[207]	TL with DNN, CNN	DoS, DDoS, Botnet, Brute Force	Accuracy, Precision, F1-score	Over 99% accuracy for large datasets
[208]	FL	DoS, Spoofing, Jamming, Blackhole	Accuracy, Precision, Recall, F1-score	99.72% accuracy, 99.70% precision
[209]	Tree-Based EL	DoS, DDoS, Fuzzy, Spoofing, Port Scanning	Accuracy, F1-score	0.965 (CICIDS2017), 0.9999 (Car-Hacking)
[210]	ML-based Trust	Data Falsification, Message Tampering	Precision, Recall, F1-score	High precision and F1-score for detecting malicious vehicles

**Table 9:** AI-based solution models for IoT applications in IIoT.

Citation	Year	IoT app	Focused Area	Ai Approches	Security Application	Advantages	Limitations
[46]	2020	IIoT	Security	ML	FDI Attack Detection	High detection accuracy, works with unlabeled data, detects unknown attacks	High computational complexity for large datasets
[55]	2020	IIoT	Security	ML	Attack Detection	High accuracy, reduced prediction time	Computationally intensive on large datasets
[211]	2020	IIoT	Security	DL	Trust Boundary Protection	High robustness against adversarial attacks, improved classification accuracy	High computational cost, requires a large dataset for training
[212]	2020	IIoT	Privacy, Security, Trust	ML and FL	Privacy- preserving	Strong privacy guarantees, enhanced security	High computational cost, complex implementation
[149]	2020	IIoT	Security, Privacy	FL,	Malware Detection	High accuracy, privacy-preserving, robust against adversarial attacks	High computational cost, complex implementation
[80]	2021	IIoT	Security, Privacy	FL	Anomaly Detection	High accuracy, privacy-preserving, decentralized model	High computational cost, complexity of implementation
[213]	2021	IIoT	Security, Privacy	FL	Intrusion Detection	High privacy, supports non-IID data, strong intrusion detection	Computationally intensive, requires complex setup
[136]	2021	IIoT	Security, Privacy	FL and DL	Anomaly Detection	High detection accuracy, privacy-preserving	Computational complexity, non-IID data challenge
[214]	2021	IIoT	Security, Trust, Privacy	FL	Intrusion Detection	Ensures fairness, high trust, privacy-preserving	High computational cost, complex implementation
[144]	2022	IIoT	Security	DL	Botnet Detection	High detection rate, low processing time	High computational complexity for large datasets
[215]	2022	IIoT	Security	ML	Intrusion Detection	Handles imbalanced data, high accuracy	Requires fine-tuning and high computational cost
[216]	2022	IIoT	Security	DL	Intrusion Detection	High detection accuracy, optimal feature selection, low processing time	High computational complexity for large datasets
[130]	2022	IIoT	Security	EL	Intrusion Detection	High accuracy, efficient feature selection, scalable for edge networks	High computational cost for large datasets
[217]	2022	IIoT	Security, Trust	TL	Trust Evaluation	High accuracy, efficient data fusion, reduced training time	High computational complexity, requires large datasets
[49]	2023	IIoT	Security	DL	IDS, Malware Detection	High accuracy, handles imbalanced data	Longer training times, high computational cost

**Table 10:** Performance of different AI algorithms for IoT security in IIoT.

Ref	Model	Attacks Types	Performance Metric Used	Results
[46]	Autoencoders	FDI	Accuracy, MSE, False Alarm	100% detection in case 1, 95% in case 2
[55]	RaNN	DoS, Data Type Probing, Malicious Control, Scan, Malicious Operation, Spying,	Accuracy, Precision, Recall, F1-Score	99.20% accuracy, 34.51 ms prediction time
[211]	Downsampler- Encoder with DNN	DDoS, Command Injection, Relay Misconfiguration, Malware Injection	Accuracy, Cross-Entropy Loss	99.20% accuracy, low cross-entropy loss
[212]	FedML	Adversarial Attacks, Data Leakage, Man-in-the-Middle	Accuracy, Latency, Privacy Budget	High accuracy, low latency, strong privacy guarantees
[149]	GAN	Adversarial Attacks, Data Poisoning, Backdoor	Accuracy, Precision, F1-Score	8% higher accuracy than existing models
[80]	FL with GRUs	Man-in-the-Middle, Ping DDoS, SYN DDoS, Modbus Query Flood	Accuracy, Precision, Recall, F1-Score	High accuracy, low false alarm rate
[213]	FedAvg, Fed+	DDoS, Backdoor, Command Injection	Accuracy, Privacy Budget	High accuracy, maintains privacy
[136]	FL with DRL	Data Breaches, Anomalous Behavior	Accuracy, Latency, False Alarm Rate	High accuracy, low latency, reduced FAR
[214]	FL	Adversarial Attacks, Model Poisoning	Accuracy, Trust Score, Reputation	High accuracy, fairness achieved, improved trust scores
[144]	Hybrid (LSTM-DNN)	Gafgyt, Mirai Botnets	Accuracy, F1-Score, Precision	99.94% accuracy, 0.066 ms detection time
[215]	XGBoost	Ransomware, DDoS, Command Injection	Accuracy, F1-Score, Precision	99.9% F1 on X-IIoTID, 99.87% F1 on TON_IoT
[216]	SDPN	DoS, U2R, R2L, and Probe Attacks	Accuracy, Precision, Recall, F1-Score	High accuracy of 99.02%, as well as superior precision, recall, and F1-score values
[130]	Stacked Ensemble	DDoS, Ransomware, Brute Force	Accuracy, Precision, F1-Score	99.7% accuracy, improved F1-score
[217]	DRL, TL	Privacy Attacks, Task Sabotage	Accuracy, FAR, MDR	99% accuracy, low FAR and MDR
[49]	Bi-LSTM, GRU	Backdoor, DDoS, DoS, Injection, Ransomware	Accuracy, F1-Score, AUC-ROC	99.99% accuracy, 0.001% error rate

**Table 11:** Summary of AI approaches used in IoT security: strengths, limitations, and future directions.

AI Approach	Application Domains	Strengths	Limitations	Future Directions
Deep Learning (DL)	Smart cities, IIoT, healthcare	High accuracy in complex tasks, automated feature extraction	High computational cost, slow training	Lightweight DL models for edge devices; energy-efficient architectures
Federated Learning (FL)	Healthcare, IoV, IIoT	Privacy-preserving, decentralized training	Communication overhead, convergence challenges	Efficient FL protocols; integration with blockchain; real-world deployments
Transfer Learning (TL)	IoV, IIoT	Reduces training time and data requirements	Domain mismatch, negative transfer risk	Cross-domain adaptation; task-specific fine-tuning for IoT
Ensemble Learning (EL)	Smart cities, healthcare, IIoT	Improves accuracy and robustness	High resource demand; complex implementation	Adaptive and lightweight ensemble strategies for constrained environments
Explainable AI (XAI)	Smart cities, healthcare	Enhances transparency and trust in ML decisions	Reduced accuracy; limited tool maturity in IoT	Domain-specific XAI models; balancing explainability and performance
Anomaly Detection (Unsupervised)	All domains	Detects unknown attacks without labeled data	High false positives; hard to evaluate accuracy	Hybrid models combining unsupervised and supervised learning
Reinforcement Learning (RL)	Resource allocation, adaptive security policies	Dynamic decision-making; environment-aware learning	Slow convergence; exploration risks	Safe and scalable RL for dynamic IoT contexts

## Data Availability

Not applicable.
